# Vaccination coverage and access among children and adult migrants and refugees in the Middle East and North African region: a systematic review and meta-analysis

**DOI:** 10.1016/j.eclinm.2024.102950

**Published:** 2024-11-22

**Authors:** Oumnia Bouaddi, Farah Seedat, Hassan Edries Hasaan Mohammed, Stella Evangelidou, Anna Deal, Ana Requena-Méndez, Mohamed Khalis, Sally Hargreaves, Middle East and North Africa Migrant Health Working Group

**Affiliations:** a Mohammed VI International School of Public Health, Mohammed VI University of Sciences and Health, Casablanca, Morocco; b Department of Public Health and Clinical Research, Mohammed VI Center for Research and Innovation, Rabat, Morocco; c Barcelona Institute for Global Health (ISGlobal, Hospital Clinic – University of Barcelona), Barcelona, Spain; d Facultat de Medicina i Ciències de la Salut, Universitat de Barcelona (UB), Barcelona, Spain; e The Migrant Health Research Group, City St George's, University of London, London, United Kingdom; f University of Gezira, Sudan; g Department of Medicine, Solna, Karolinska Institutet, Solna, Sweden; h CIBERINFEC, ISCIII - CIBER de Enfermedades Infecciosas, Instituto de Salud Carlos III, Centro de Investigación Biomédica en Red de Enfermedades Infecciosas, Madrid, Spain; i Higher Institute of Nursing Professions and Health Techniques, Rabat, Morocco

**Keywords:** Vaccination, Migrants, Refugees, Vaccine-preventable diseases, Immunisation, Middle East and North Africa

## Abstract

**Background:**

The Middle East and North African (MENA) region is a major global hotspot for migration with more than 40 million migrants, who may be an under-vaccinated group because of barriers to vaccination within countries of origin, transit, and destination. We systematically synthesised the evidence on coverage, acceptance, drivers of uptake, and policies pertaining to vaccination for children and adult migrants in the region, in order to explore tailored interventions for these groups.

**Methods:**

We searched six databases (including Medline, Embase) for peer-reviewed literature, and other websites (including WHO, IOM, ministries of health) for grey literature on coverage, acceptance, drivers of uptake and policies for any vaccination in migrants in the MENA region from between 2000 and 27 August 2024 in any language. We included studies reporting primary data on coverage, acceptance, and drivers of uptake, and any relevant articles on policies. We defined migrants as individuals who move away from their place of habitual residence, within or across international borders, temporarily or permanently. Studies without disaggregated migrant data were excluded. Primary outcomes were coverage (% individuals receiving ≥1 doses of any vaccine) and acceptance (% individuals accepting any vaccine). We separately synthesised data on children (<18 years) and adults (≥18). Estimates were pooled using a random-effects meta-analysis where possible or narratively synthesised, and drivers of uptake were synthesised using the WHO Behavioural and Social Drivers model. PROSPERO protocol: CRD42023401694.

**Findings:**

We identified 6088 database and 282 grey literature records and included 55 studies and 1,906,975 migrants across 15 countries (including mostly refugees in the Middle East and expatriates in Gulf Cooperation Council countries). COVID-19 vaccination was reportedly provided free of charge to migrants in all countries whereas childhood vaccinations were reportedly provided to migrant children in seven countries. However, for adolescents and adults, there were wide variations across countries, and we found no policies relating to catch-up vaccination. Coverage for childhood vaccination amongst migrants was reportedly low, with only 36.0% of 589 migrant children fully vaccinated according to national schedules (95% CI 35.0%–43.0%, *I*^2^ = 67%; data from migrants in Lebanon, Morocco, Sudan). Likewise, data on specific routine vaccines in children was generally low: measles containing vaccines (MCV): MCV dose 1 63.9%–66.9%; MCV dose 2 25.4%–85.6%; oral polio vaccine (OPV): OPV dose 3 65.1%–76.4%; diphtheria, tetanus and pertussis (DTP) containing vaccines: DTP dose 1 81.8%–86.7%; DTP dose 3 59.7%–76.6%). Drop-out rates across all routine vaccines for subsequent vaccine doses ranged from 12.4 to 38.5%, suggesting that migrants face a range of barriers to vaccine uptake beyond the first dose, that need to be better considered when designing interventions. For adults, we found eleven studies on coverage (including 9 on COVID-19) showing that COVID-19 vaccination coverage ranged 33.5–84.8% in migrants and 25.0–59.0% in host populations. Drivers of uptake of childhood vaccination in migrants included limited availability of vaccines and vaccination personnel, communication and administrative barriers, financial difficulties, lack of caregiver knowledge about services, and concerns expressed by caregivers around safety and benefits. For adults, drivers were mainly related to the COVID-19 vaccine and included concerns around safety, quality, side effects, and mistrust in vaccines and the systems that deliver them.

**Interpretation:**

Migrants have unique risk factors for under-immunisation in the MENA region and have low vaccination coverage despite some level of entitlement to services. Data on vaccination coverage, drivers of uptake and policies for migrants in the MENA region is limited to small-scale studies among accessible groups, mostly focusing on COVID-19 compared to routine childhood and adult vaccination. There is an urgent need to strengthen data collection to better understand coverage across different migrant groups, ages, and MENA countries, especially on adult and catch-up vaccinations for routine immunisations, and develop innovative co-designed strategies to address specific drivers of vaccine uptake among this group.

**Funding:**

La Caixa, LCF/PR/SP21/52930003.

**Research in context**

**Evidence before this study**

Forty million migrants live in the Middle East and North Africa (MENA) region, including 24.1 million workers, over 6.9 million refugees (UNHCR 2023), and 6 million Palestinian refugees (UNRWA 2023). Globally, migrants are considered to be under-vaccinated due to barriers to vaccination services and non-systematic inclusion in national vaccination policies. Before this study, a global meta-analysis by Cherri et al. of 7,375,184 participants across over 10 countries showed 50% lower vaccination coverage in migrant adults and children than host populations. In the MENA, little data exists on migrant inclusion in vaccination programs or coverage to guide interventions. The International Organization for Migration (IOM) reported that migrants were included in National COVID-19 Deployment and Vaccination plans in the MENA region, however, these strategies were not always implemented as planned and it is unclear to what extent different migrant groups were included in practice. Additionally, outbreaks of polio, measles, and other vaccine-preventable diseases (VPD) among migrants and refugees in war-torn areas like Syria, Lebanon, and Iraq suggest under-vaccination in these groups.

**Added value of this study**

This is the first large systematic review and meta-analysis on coverage, acceptance, policies, and drivers of vaccination among migrants in MENA. The research thoroughly examines peer-reviewed and grey literature, offering insight into migrants’ vaccination coverage and access in the region. We reviewed data from 15 MENA countries, covering 1,906,975 migrants (mainly refugees in the Middle East, like Lebanon and Jordan, or expatriates in GCC countries). Although migrants have some entitlement to services, there is low vaccine schedule completion and low coverage for key routine childhood vaccinations (e.g. measles, diphtheria, tetanus, pertussis, polio), and adults are poorly considered in catch-up vaccination initiatives for missed vaccines, doses, and boosters. Adult coverage data was limited, mainly showing inconsistent COVID-19 vaccination levels. For both child and adult vaccination, persistent access barriers exist, conceptualised into a drivers of vaccine uptake framework for migrants in the MENA region. These findings highlight the need to strengthen vaccination monitoring for migrants and address the barriers they face.

**Implications of all the available evidence**

Migrants in the MENA may have low vaccination coverage, and their needs have not been prioritised in research. Expanding the evidence on coverage, policies, and drivers of uptake for both childhood and adult vaccination for MENA migrants is essential. More studies are needed on adult and catch-up vaccination for all outcomes, as current research focuses mainly on COVID-19. Large-scale studies on life-course vaccination, disaggregated by migrant subgroups, are needed. Meanwhile, to meet global immunization goals, strategies must ensure universal, equitable access to life-course vaccination for migrants, even in inclusive-policy countries. Host countries should tailor services to address specific drivers identified in this review, using participatory, co-design approaches with migrant communities and integrating migrants into health information systems for better vaccination monitoring.

## Introduction

Globally, access to vaccination for migrant populations (defined as any individuals who move away from their usual place of residence between or within a country)^1^ is a critical aspect in the prevention and control of infectious diseases and the improvement of morbidity and mortality outcomes.^2^ Indeed, the World Health Organization's Immunization Agenda 2030 (WHO IA2030) calls for the equitable and universal access to life-course vaccination for all population groups globally.^3^ However, some migrant groups (e.g. asylum seekers, undocumented migrants) are found to have lower vaccination rates compared to non-migrant populations.^4^^,^^5^ In Europe, a study among 16,701 migrant children reported lower vaccine uptake for all recommended childhood vaccines compared to Danish-born children particularly for diphtheria, tetanus, and pertussis (DTP) containing vaccine (Hazard ratio = 0.50; 95% CI 0.48–0.51).^6^ Similarly, a global systematic review in over 15 countries found that adult and child migrants had lower vaccination coverage compared to non-migrants (Odds ratio 0.50; 95% CI 0.37–0.66).^7^ Many migrants experience vaccine hesitancy and face individual and systemic barriers in accessing routine and catch-up vaccination services, including language and administrative issues, legal and financial hardships, and discrimination and margainsalisation.^2^^,^^8^ Likewise, their inclusion in national vaccination policies is not systematic.^2^ This lack of access to vaccination combined with inadequate living conditions particularly in shelters, detention and reception centers, results in migrants experiencing a higher burden of vaccine-preventable diseases (VPD), with several VPD outbreaks reported among this group.^9^^,^^10^

The Middle East and North African (MENA) region hosts more than 40 million migrants.^11^^,^^12^ This migration influx is only increasing across the region, given the mass international and internal displacement in Gaza and Sudan compounded with protracted conflicts in the Syrian Arab Republic, and Yemen.^13^ In fact, the MENA region is home to half of the world's refugees,^13^ most of whom reside in neighbouring countries such as Jordan and Lebanon, which have the highest per capita number of refugees.^14^^,^^15^ Despite the high migration influx, vaccination coverage in migrants residing across this region is unclear. There is evidence of significant disparities in access to healthcare services, including vaccination, for vulnerable populations in fragile and conflict-affected countries (e.g. Iraq and Syria), as well as neighbouring countries (e.g. Jordan and Lebanon) as a result of the disruption of healthcare systems.^11^^,^^16^^,^^17^ For example, in 2022, coverage for the third dose of DTP (often used as a proxy indicator for the reach of immunization programmes as opposed to DTP1 which is used as an indicator for unvaccinated or zero-dose children^18^) was below the 90% global target in some MENA countries such as Syria (65.4%) and Yemen (89%).^19^ Yet, it is unclear how migrant populations are impacted or what the vaccination coverage in migrant population is across the region. Recurring outbreaks of VPDs have been documented among migrant children in humanitarian settings particularly among those residing in settlements, collective shelters, camps, and in remote and conflict prone areas.^2^^,^^9^^,^^20^ For instance, the Syrian crisis resulted in several outbreaks of hepatitis A, cholera, measles, and polio, affecting both internally displaced populations (IDPs) and neighboring countries hosting refugees such as Lebanon, Turkey, Jordan, Iraq, and Egypt.^21^, ^22^, ^23^, ^24^, ^25^ This suggests that migrant children in the region may be under-vaccinated. In adults, during the COVID-19 pandemic, some countries such as Jordan rapidly included migrants in the national COVID-19 vaccination campaign, however, some migrant groups in the region were excluded from vaccination efforts.^26^

Overall, there is a lack of understanding of the vaccination coverage in migrant children and adults across and between countries in the region. Likewise, there is limited understanding of the vaccination policies pertaining to migrants across countries in the region and the volume and quality of data reported on the drivers of vaccine uptake in migrants in the region. This lack of information poses significant challenges to monitoring the health of these groups and identifying approaches to improve access and achieve global vaccination targets. Therefore, we conducted a systematic review to synthesise the evidence on (1) vaccination coverage and acceptance (2) vaccination policies and (3) drivers of vaccination uptake pertaining to migrant populations in the MENA region.

## Methods

This systematic review and meta-analysis is reported according to the PRISMA 2020 guidelines.^27^ We registered the protocol with PROSPERO [CRD42023401694] and published it.^12^

### Inclusion and exclusion criteria

We included studies with primary data published in any language after the year 2000 on 1) coverage and acceptance 2) policies, and 3) drivers of vaccination among migrant populations (definitions in Box 1) in 16 MENA countries (Algeria, Bahrain, Egypt, Iraq, Jordan, Kuwait, Lebanon, Libya, Morocco, Occupied Palestinian Territories, Oman, Qatar, Saudi Arabia, Sudan, Syrian Arab Republic, Tunisia, United Arab Emirates, and Yemen).^28^ For policy outcomes, we also included secondary research studies such as reviews, perspectives, letters to the editor and commentaries. We included papers discussing vaccines for the following VPDs: Cholera; Dengue; Diphtheria; Hepatitis; *Haemophilus influenzae* type b (Hib), Human papillomavirus (HPV), Influenza, Measles, Meningococcal diseases, Mumps, Pertussis, Pneumococcal disease, Poliomyelitis, Rabies, Rubella, Rotavirus, Tetanus, Typhoid, Tuberculosis, Varicella, and COVID-19. We excluded studies if they did not meet the key definitions for migrant, VPD, or included countries, or where studies did not disaggregate data for migrants, or were published before 2000 to keep findings relevant to recent migrant population flows and policy.

Box 1 **Definitions**

**Migrant:** An umbrella term, not defined under international law, reflecting the common lay understanding of a person who moves away from his or her place of usual residence, whether within a country or across an international border, temporarily or permanently, and for a variety of reasons. The term includes a number of well-defined legal categories of people, such as migrant workers; persons whose particular types of movements are legally-defined, such as smuggled migrants; as well as those whose status or means of movement are not specifically defined under international law, such as international students.^1^

**Refugees:** defined by the 1951 Refugee Convention as individuals who, due to a well-founded fear of persecution based on race, religion, nationality, social group, or political opinion, are outside their country of nationality and unable or unwilling to seek protection from it.^30^

**Asylum-seekers:** individuals seeking international protection whose claims for refugee status have not yet been decided by the host country.^31^

**Internally Displaced Populations (IDPs):** people forced to flee their homes due to conflict, violence, human rights violations, or disasters, but who remain within their country's borders.^31^

**Vaccination coverage:** refers to the percentage of individuals who have received one or more doses of any vaccine, or the complete recommended series regardless of age. This determination is made through the examination of vaccination cards, recall, or a combination of both, in relation to the total number of a specific population under study. Alternatively, the calculation may be based on the total target or eligible specific population within the country where the study is conducted.

**Fully-vaccinated:** Refers to individuals who have received the entire recommended series of vaccines according to the vaccine schedule in the country of the study, irrespective of whether the vaccinations were administered in a timely manner or not, and irrespective of how status was assessed (card, recall or both).

**Vaccination acceptance:** The percentage of people who accept vaccination.

**Vaccine hesitancy:** The percentage of people who question, delay or refuse vaccination even when safe vaccines are available.

### Search strategy

We searched Medline, Embase, CINAHL, Web of Science Core Collection, Index Medicus of the Eastern Mediterranean Region and QScience for articles published between 2000 and 14 February 2023 in any language, combining free text and subject heading terms for migrant, vaccination or VPDs, and MENA (see Table S1 in Supplementary Material for full search strategy). We updated the search on August 27, 2024, to identify additional eligible studies published since the initial search in February 2023. We also performed an extensive search of grey literature sources from the following websites: International Organization for Migration websites (IOM Global Migration Data Analysis Center, IOM MENA, IOM Displacement Tracking Matrix, IOM Publication platform), UNHCR, UNDESA, UNRWA, WHO, UNICEF, GAVI, MIPEX, Migration Governance Index, and ministry of health websites for each country included in the MENA region. Records were imported into the web-based application Rayyan,^29^ duplicates were deleted and two reviewers (OB, HE) independently assessed all titles, abstracts, and full texts. Any disagreements were discussed with a third reviewer (FS) where necessary. The two reviewers also independently reference-checked all included studies and relevant systematic reviews. We also contacted experts for additional references, particularly regarding policies.

### Data extraction

Two reviewers (OB, HE) independently extracted the following pre-defined data using a form which was piloted and refined: Information about the studies (location, year of study, study design, study population (migrant and host population), vaccine(s), vaccination type (e.g. childhood, COVID-19, adult, catch-up vaccination), outcome definitions, and results (coverage, acceptance, policies, drivers of uptake). All discrepancies were resolved by consensus with input from FS, SH, ARM.

### Quality appraisal

Two reviewers (OB, HE) independently appraised the quality of studies using Joanna–Briggs Institute (JBI) tools^32^ for cross-sectional studies. The checklist contains a total of 8 assessment criteria. Every criterion was given a rating of ‘yes’, ‘no’, ‘unclear’ or ‘not applicable’.^33^ Grey literature records were appraised using the AACODS checklist^34^ which has been widely use to assess the quality of grey literature. This checklist comprises six domains: Authority; Accuracy; Coverage; Objectivity; Date; and Significance. Each domain outlines guiding questions and is assigned a rating of ‘yes’, ‘no’, or ‘unclear’. Studies were not excluded based on quality, but the results of quality assessments contributed to the analysis and the discussion. Studies were judged to be at risk of bias overall if at least one domain was at high risk, while studies with at least one domain rated as unclear risk of bias were judged to be unclear risk of bias overall. Studies at low risk of bias for all domains were judged to be low risk overall.

### Data analysis

For coverage, we only performed meta-analysis where there was sufficient volume and homogeneity of data. For volume, we considered a minimum of three data points as the threshold to conduct the meta-analysis. For homogeneity, we considered population type, outcome measurements, and only pooled studies with similar outcome definitions, where coverage was measured by card for most participants. We did not combine different individual vaccines, pool data on adults and children, or include post-vaccination campaign data. Where these criteria applied, we performed random effects meta-analysis of a single proportion using the Metaprop function in R software version 4.3.0 (2023-04-21)^35^ to calculate the pooled proportion and corresponding 95% confidence intervals (CI). For studies reporting pre- and post-campaign data, we reported both but we only pooled pre-campaign data. We assessed heterogeneity between studies using forest plots and the *I*^2^ statistic. Where possible, we performed sensitivity analyses including only a specific type of migrants (i.e. refugees only). For individual studies where 95% CIs were not reported, we calculated binomial 95% CI using the binom.test function in R.

For studies on coverage where there was insufficient volume or homogeneity, and for the other outcomes, we narratively synthesised data in text, tables, and figures. Data on drivers of uptake were narratively synthesised using the Behavioural and Social Drivers (BeSD) of Vaccination framework developed by the WHO.^36^ This framework comprises four domains which affect vaccine uptake—thoughts and feelings, motivation, social processes and practical accessibility issues.

### Role of funding source

The funders of this study played no part in its design, data collection, analysis, interpretation, report writing, or the decision to publish.

## Results

### Characteristics of included studies

**Fig. 1:**
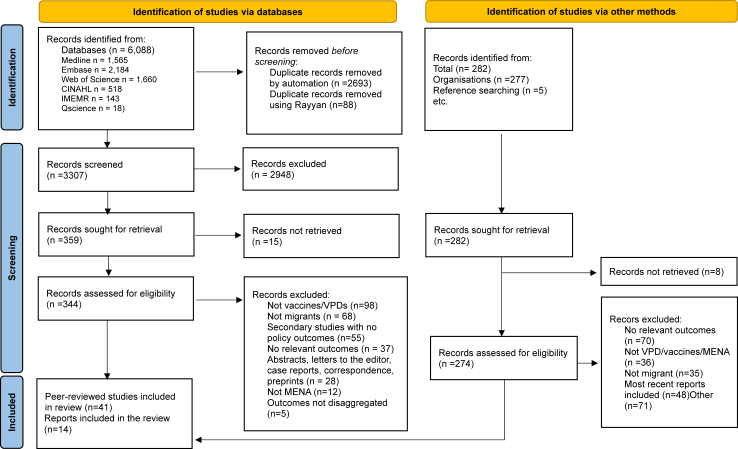
**PRISMA flowchart**.

A total of 6088 database and 282 grey literature records were identified and screened for eligibility. Of these, 344 database records and 274 grey literature records were included for full-text screening. Fifty-five studies met our inclusion criteria for the systematic review including 41 peer-reviewed studies and 14 grey literature reports (see Fig. 1 and Tables 1 and 2). Twenty-two studies reported on vaccination coverage, including 1,906,975 migrants,^38^, ^39^, ^40^, ^41^, ^42^, ^43^, ^44^, ^45^, ^46^, ^47^, ^48^, ^49^, ^50^, ^51^, ^52^, ^53^, ^54^, ^55^, ^56^, ^57^, ^58^, ^59^ 8 on vaccine acceptance or hesitancy,^40^^,^^49^^,^^55^^,^^60^, ^61^, ^62^, ^63^, ^64^, ^65^ 25 on policies,^66^, ^67^, ^68^, ^69^, ^70^, ^71^, ^72^, ^73^, ^74^, ^75^, ^76^, ^77^, ^78^, ^79^, ^80^, ^81^, ^82^, ^83^, ^84^, ^85^, ^86^, ^87^, ^88^, ^89^, ^90^, ^91^ and 14 on drivers of uptake including 17,800 migrants.^40^^,^^42^^,^^43^^,^^45^^,^^47^^,^^49^^,^^56^, ^57^, ^58^^,^^60^, ^61^, ^62^, ^63^^,^^92^^,^^93^ We found studies on 15 countries; majority were in the Middle East (Gulf Cooperation Council n = 22; Lebanon n = 9) and eight were in North Africa. Most studies were cross-sectional (n = 33). Twenty-four studies reported on refugees, 17 on expatriates or “non-nationals”, and six on IDPs.

**Table 1:** Study characteristics for vaccination coverage, acceptance and drivers of uptake.

Author, year Country	Study design	Study duration	Study setting	Vaccines	Study population	Sample size	Outcome(s)	Risk of Bias score
Alabdulla M. 2021^1^ Qatar	Cross–sectional	October 15–November 15, 2020	Online local newspapers, and across the social media platforms of the Hamad Medical Corporation	COVID–19	Adult residents of Qatar aged 18–65 years old	914 Qatari 6907 Non–Qatari	Acceptance	At–risk
AlAwadhi E. 2021^2^ Kuwait	Cross–sectional	NR	Online via social media platforms	COVID–19	Citizens and non– citizens aged 18–74 years living in Kuwait during COVID–19 pandemic	5651 Kuwaiti 1590 Non–Kuwaiti	Acceptance	At–risk
Al–Hatamleh 2022^3^ Jordan	Cross–sectional	October 16, 2021–March 22, 2022	Refugee camp	COVID–19	Adult Palestinian refugees in Jerash camp and Jordanian citizens who have not experienced life in a refugee camp	501 Refugees 491 Citizens	Coverage Drivers Acceptance	Unclear
Andrade G. 2022^4^ UAE	Cross–sectional	NR	NR	COVID–19	UAE residents who have not received the COVID–19 vaccine yet	63 Emirati 220 Arab Expatriate 112 South–east Asian 31 Other	Acceptance Drivers	At–risk
Barry M. 2021^5^ Saudi Arabia	Cross–sectional	December 27, 2020–January 3rd 2021	Online via social media platforms and email lists	COVID–19	HCWs in Saudi Arabia during the COVID–19 pandemic	736 Expatriates 322 Saudis	Coverage	At–risk
Elhadi F. 2015^6^ Sudan	Cross–sectional	May–October 2009	IDP settlements in Khartoum	MCV	Children (12–23 months)–caregivers in households in IDP settlements	210 IDP children	Coverage Drivers	At–risk
Lam E. 2017 ^7^ Iraq	Cross–sectional	December 14–16, 2015	Households in refugee and IDP camps in Iraq's governorates that were targeted during the 2015 campaign	OCV	Individuals aged 1 to <15 years old living in households in refugee and IDP camps in Iraq's governorates that were targeted during the 2015 campaign	5007 Refugees and IDPs	Coverage Drivers	At–risk
Mansour Z. 2019^8^ Lebanon	Cross–sectional	December 2015–June 2016	Households	All vaccines in the NIP	Children aged 12–59 months and caregivers in all of Lebanon except the district of Nabatieh, irrespective of their nationality	7136 Lebanese 2179 Syrian refugees	Coverage Drivers	Low
Kmeid M. 2019^9^ Lebanon	Cross–sectional	July 2017–February 2018	nurseries, schools, summer camps, waiting rooms of paediatric clinics, and dispensaries	All vaccines in the NIP	Lebanese and Syrian refugee children aged 0–15 years old	83 Syrian refugees 488 Lebanese	Coverage	Low
Rossi R. 2016^10^ Lebanon	Cross sectional	First survey on the 20th March 2015 and the post–vaccination survey on the 27th October 2015	household (including collective shelters)	All vaccines in the NIP	Lebanese and refugee children aged 12–59 months	Lebanese 127/118 (1st—2nd survey) Syrian refugees 83/92	Coverage	At–risk
Ismail I. 2014^37^ Sudan	Cross–sectional	March 22nd–March 28th, 2010	Households in Nyala locality (rural urban and IDP camps)	All vaccines in the NIP	Children aged 12–23 months (rural, urban and IDP camp)	157 Urban 35 Rural 21 IDPs camps	Coverage	At–risk
Alawieh A. 2017^11^ Lebanon	Cross–sectional	Before and after the polio immunization campaign following the outbreak in November 2013	Surveillance system of the Lebanese Ministry of Health	OPV	Adult and child Lebanese population and syrian and refugee population	5,882,562 Lebanese 1,435,840 Syrian refugee 455,000 Palestinian refugee	Coverage	Unclear
Shehab M. 2021^12^ Kuwait	Cross–sectional	1 June 2021–31 October 2022	University hospital, a tertiary care inflammatory bowel disease center	COVID–19	Adult patients with Inflammatory Bowel Syndrome (IBD) receiving biologic therapies	201 Citizens 79 Expatriates	Coverage	Unclear
Toraimbe S. 2021^13^ Morocco	Cross–sectional	March 29th–September 24th, 2020	NR	All vaccines in the NIP	Sub–saharan migrant children–mother dyads (children under 5 years old)	402 Sub-saharan African migrant mother–child dyads	Coverage Drivers	At–risk
Nizam A. 2022^14^ UAE	Cross–sectional	April 2021–June 2021	Web–based, Universities	COVID–19	Full–time students enrolled at either a private or public higher education institution in the UAE, in a program with English as the language of instructions.	33 UAE National 352 Expatriates	Drivers	At–risk
Reagu S. 2022^15^ Qatar	Cross–sectional	Between 15 October and the 15 November 2020	Online through online local newspapers, and across the social media platforms	COVID–19	Adults Qatar population (general public and healthcare workers)	653 Qatari 4687 Non–Qatari	Acceptance	At–risk
Talafha Q. 2022^16^ Jordan	Cross–sectional	January–March 2022	Web–based through social media	COVID–19	Refugees over 18 years old in the Zaatari refugee camp	230 Syrian refugees	Acceptance Drivers	Low
Khan A. 2008^17^ Saudi Arabia	Cross–sectional	2005	Community (shops, restaurants and mosques)	Hepatitis B	Blue collar expatriate workers both skilled and non–skilled	665 blue collar expatriate workers	Coverage	At–risk
Zeid B. 2022^18^ Lebanon	Cross sectional	September 2020–March 2022	Households	COVID–19	All households in Lebanon that received assistance from the Norwegian Refugee Council and had an older Syrian refugee (<50 years old)	2906 Syrian refugees 1822 outside informal settlements 1084 inside formal settlements	Acceptance Drivers	At–risk
Ministry of Health 2019^19^ Saudi Arabia	National survey report	2019	Households	MMR, DTwP	Children	88 Saudi 6 Non–Saudi	Coverage	At–risk
UNHCR 2022^20^ Jordan	Monthly report	2022	Camps and nationwide	COVID–19	Syrian refugees in camps and Jordan population	Syrian refugees	Coverage	At–risk
UNHCR 2022^21^ Lebanon	Monthly report	2022	Camps and nationwide	COVID–19	Syrian refugees in camps and Jordan population	Syrian refugees	Coverage	At–risk
Farag N. 2020^22^ Jordan	Cross-sectional	May 2016	Households in High–Risk Areas (HRAs) and two refugee camps in Jordan (Azraq and Zaatari)	OPV and IPV	Children aged 6–59 months and caregivers living in Jordan's HRAs and two refugee camps	HRAs: 375 Jordanian 104 refugees Camps: 276 Syrian refugees	Coverage Drivers	At–risk
Jaber S. 2005^23^ Saudi Arabia	Cross-sectional	Year 2004	Preschool, Kindergarten, primary, elementary, secondary schools located at different urban regions	MCV	School–aged children 1–14 years old	298 Saudi 229 Non–Saudi	Distribution Coverage	At–risk
Al–Kuwari MG 2011^24^ Qatar	Cross-sectional	2007	Measles surveillance system of the public health department of the National Health Authority in Qatar	MCV	Reported (confirmed) measles cases in 2007	100 Qatari 241 Non–Qatari	Distribution Coverage	At–risk
Ali M. 2024^25^ Jordan	Cross-sectional	first 2 weeks of December 2021	Primary healthcare centres, schools, public areas and lounges	COVID-19	Refugees and migrants aged 12 years and above residing in Jordan regardless of migration status	259 Palestinians 200 Syrians 93 Non-arabs 84 Arabs	Coverage Drivers	At-risk
Gubari M. 2023^26^ Iraq	Cross-sectional	April–May 2022	Households	COVID-19	The general population, IDPs, and refugees aged 12 years or older	3519 from host communities 428 IDPs 617 refugees	Coverage Drivers	At-risk
Dalky A. 2024^27^ Jordan	Cross-sectional	11 October 2022–15 April 2023	Two refugee camps	COVID-19	Pregnant and lactating women aged 18 years and above	385 Syrian refugee women	Coverage Acceptance	At-risk
Aschore M. 2024^28^ Libya	Cross-sectional	April–July 2021	Households	COVID-19	Forcibly displaced people	1448 migrants 2019 refugees	Coverage Drivers	At-risk
Abdulhaq B. 2024^29^ Jordan	Cross-sectional		Refugee camps and communities	All routine vaccines	Syrian refugees	332 refugees 167 inside camps 165 outside camps	Drivers	At-risk

NIP, National Immunization Programme; MCV, Measles containing vaccines; OPV, Oral polio vaccine; IPV, Inactivated polio vaccine.

**Table 2:** Study characteristics for vaccination policies.

Author/organization, year Country	Study design or type of publication	Policy level	Vaccines	Target population	Risk of bias
Tazyeen S. 2022^30^ Middle East	Perspective	National	COVID–19	Foreign workers with a valid emirates ID	Low
Rahman M. 2022^31^ Gulf countries	Narrative review	Regional	COVID–19	Migrant workers	Low
Assiri A. 2021^32^ Saudi Arabia	Commentary	National	COVID–19	Expatriates	Low
Habersky E. 2021^33^ EMR	Narrative review	National	COVID–19	Refugees registered with UNHCR	Low
Ministry of Health 2021^34^ Tunisia	Strategy document	National	COVID–19	Migrants, refugees and individuals with irregular status	Low
Suliman D. 2021^35^ UAE	Commentary	National	COVID–19	Citizens and residents	Low
UN Network for Migration 2021^36^ Bahrain	Policy brief	National	COVID–19	Citizens, residents and undocumented migrants	Low
WHO 2022^38^ Bahrain	Case study	National	COVID–19	Residents, regardless of nationality, residential status, or ethnicity	Low
Ministry of Public Health 2021^39^ Lebanon	Guidelines	National	COVID–19	All residents regardless of nationality	Low
IOM 2021^40^ Egypt	Report	National	COVID–19	Migrants and refugees	Low
Jawad J. 2011^41^ Bahrain	Cross sectional, descriptive	National	MCV	Citizens and residents	Unclear
Riccardo F. 2012^42^ EpiSouth including North Africa (Algeria), Tunisia, Morocco)	Cross–sectional study	National	NS	Mobile communities (regular, irregular and nomadic)	Low
Giambi C. 2017^43^ Mediterranean Basin and Black Sea	Cross–sectional study	National	All vaccines in the NIP	Newly arrived migrants	Low
Ministry of Health 2023^44^ Syria	Epi bulletin	National	Polio	IDPs and returnee migrants	Low
Gulf Health Council 2021^45^ Gulf countries	Guidelines	Regional	All vaccines in the NIP	Expatriates coming to GCC states for work or residence	Low
Gulf Health Council 2021^46^ Gulf countries	Guidelines	Regional	Polio, MMR 1 and 2, Meningococcal vaccine, COVID–19	Expatriates coming to GCC states for work or residence	Low
WHO^47^ EMR	Report	National	All vaccines in the NIP	All migrants visiting primary care centers	Low
IOM 2021^48^	Report	National	All vaccines in the NIP	Refugees and asylum seekers, regular and irregular migrants, IDPs	Low
Ministry of Foreign Affairs 2017^49^ Morocco	Report	National	All vaccines in the NIP	All migrants regardless of status	Low
Santus D. 2023^50^ Morocco	Cross-sectional qualitative study	National	COVID-19	All migrants and refugees	Low
Alahmad B. 2023^51^ Gulf countries	Commentary	Regional	COVID-19, Flu and Pneumococcal vaccines	Non-nationals	Low
Honein-AbouHaidar G. 2024^52^ Lebanon	Cross-sectional mixed-methods study	National	All routine vaccines	Syrian refugees	Low
Rahman MM. 2023^53^ Qatar	Narrative review	National	COVID-19	Migrant workers	Low
Chen S. 2024^54^ Gulf countries	Narrative review	Regional	COVID-19	Residents	Low
Ismail S. 2023^55^ Lebanon	Cross-sectional qualitative study	National	All routine vaccines	Syrian refugees	Low

NIP, National Immunization Programme; IDPs, Internatlly-displaced populations; MMR, Measles, Mumps and Rubella.

For all outcomes, thirty studies were on COVID-19, eleven on 9 other vaccines (Measles containing vaccines (MCV), DTP containing vaccines, Oral and Inactivated Polio Vaccine (OPV/IPV), Hepatitis B (HepB) vaccine, Rotavirus vaccine (RV), Meningococcal vaccine, Oral Cholera Vaccine (OCV), Hib vaccine, and Pneumococcal vaccine), and 13 studies covered all routine vaccines in the National Immunisation Programme (NIP) (see Tables 1 and 2). Of the 22 studies reporting vaccination coverage, 11 studies were among 8737 migrant children^38^, ^39^, ^40^^,^^42^, ^43^, ^44^, ^45^^,^^47^^,^^52^, ^53^, ^54^ and 8 studies were among 8125 adults^46^^,^^48^, ^49^, ^50^, ^51^^,^^61^ Only four studies on 589 migrants could be meta-analysed.^38^^,^^47^^,^^52^^,^^53^ Below, we report the results separately for children and adults.

### Quality appraisal

Only five cross-sectional studies assessed using JBI were at low risk of bias for all domains, four studies were at unclear risk of bias, and 24 studies at some risk of bias (Tables 1 and 2). The most frequent risks of bias were observed in domains associated with the definition of exposure (n = 11), the outcomes not measured in a reliable (n = 12) or standard way across all participants (n = 6), and the description of the settings not being provided in sufficient details (n = 6). Overall, studies at risk or unclear risk of bias did not provide a clear definition of migrant, used records to assess coverage for some participants and recall for the rest, or did not have sufficient information about study settings. Grey literature records assessed using the AACODS tool (n = 14) had sufficient quality overall as majority of the papers were simply describing policies.

### Completion of childhood vaccination schedules

**Fig. 2:**
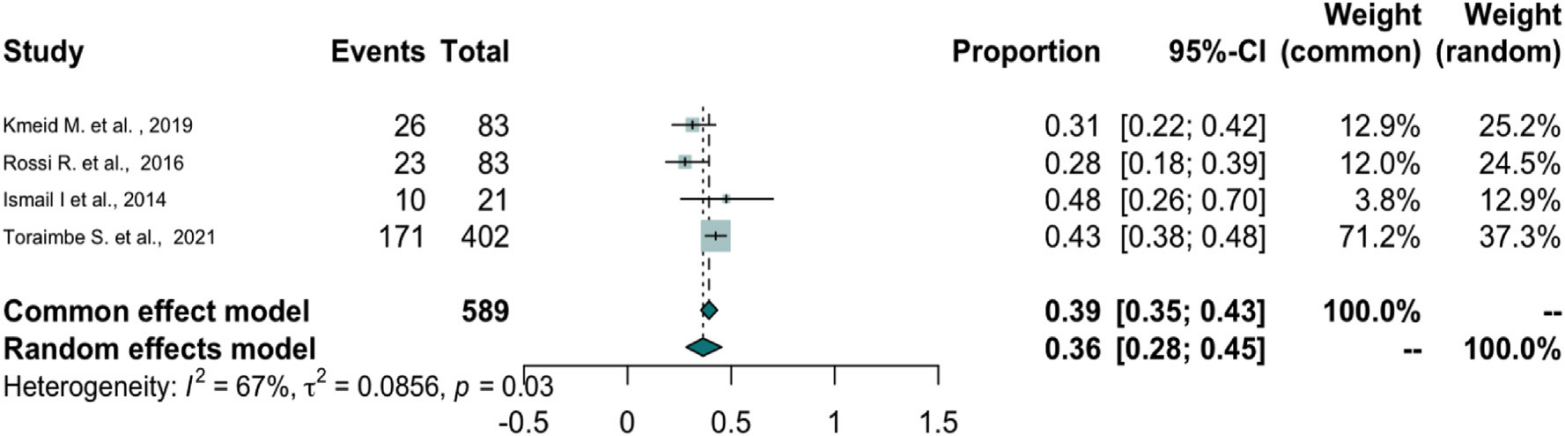
**The forest plot of the pooled estimates of fully–vaccinated migrant children**.

Four studies assessed whether migrant children were ‘fully vaccinated’ according to the countries' national schedules.^38^^,^^47^^,^^52^^,^^53^ The percentage of fully vaccinated migrant children was 27.7–48.0% across four studies^38^^,^^47^^,^^52^^,^^53^ and the percentage of fully-vaccinated host children was 36.2–71.6% across three studies.^38^^,^^52^^,^^53^ When pooled together, the percentage of fully vaccinated migrant children was 36.0% (N = 589, 95% CI 0.35–0.43, *I*^2^ = 67%) (Fig. 2—forest plot).^38^^,^^47^^,^^52^^,^^53^ We performed a sensitivity analysis only including three studies of migrants residing in community settings and removing one study conducted among IDPs in camps,^53^ and the pooled estimate remained similar (0.35, 0.26–0.45, *I*^2^ = 67%).

Two studies assessed the odds of being fully vaccinated in migrant vs host children, finding that there was no statistical difference between the groups (see Table S2–Supplementary material for AOR and factors adjusted for).^38^^,^^52^ One study assessed socio-demographic predictors of vaccine completion among migrant children finding that mother's education and professional status were associated with the child being fully vaccinated^47^ (see Table S3–Supplementary Material for ORs).

### Childhood vaccination coverage for key individual vaccines

Nine studies assessed coverage for nine individual vaccines (see Table 3).^38^, ^39^, ^40^, ^41^, ^42^, ^43^, ^44^, ^45^^,^^54^ Five of these compared migrants to host children,^38^^,^^39^^,^^41^^,^^43^^,^^44^ generally finding that migrants had a lower coverage across all vaccines, although coverage was low in both populations for some vaccines. For example, coverage for the first and second doses of MCV (MCV1 and MCV2) among migrants was low (MCV1 63.9%–86.6%, MCV2 25.4–85.6%) across 3 studies. Regarding full measles vaccination (two doses), it was low among both migrants and host in one study (32.5% vs 38.6%),^38^ high among both migrants and host in another study (99% vs 98%),^54^ and low in migrants and high host in one study (86.1% vs 100%).^44^

**Table 3:** Vaccination coverage by vaccine and by dose among migrant and host populations.

Author, year Country	Setting	Study population	Migrant^g^ N (%)						Host N (%)					
			Dose 1	Dose 2	Dose 3	Dose 4	All doses	Any dose	Dose 1	Dose 2	Dose 3	Dose 4	All doses	Any dose
**Measles containing vaccines (MCV, MMR)**														
Rossi R. 2016^10^ Lebanon	Households and collective shelters	210 Lebanese and refugee children aged 12–59 months	63.9 (53.5–74.2)/91.3 (85.5–97.1)^i^	25.4 (15.0–35.8)/59.2 (49.0–70.0)^i^			32.5 (22.5–42.6)/62.0 (52.0–71.9)^i^		66.9 (58.7–75.1)/87.3 (81.3–93.3)^i^	35.6 (26.3–45.0)/51.1 (41.0–61.2)^i^			38.6 (30.1–47.0)/60.2 (51.3–69.0)^i^	
Ministry of Health 2019^19^ Saudi Arabia	NR	88 Saudi and 6 Non–Saudi children	86.6 (35.9–99.5)	85.6 (35.9–99.6)			86.1		100	100			100	
Al–Kuwari MG 2011^24^ Qatar	Measles surveillance system	341 confirmed measles cases 100 Qatari, 241 Non–Qatari)						16.8						18.0
Jaber S. 2005^23^ Saudi Arabia	Schools	289 Saudi and 229 non-Saudi school–aged children 1–14 years old					99.0						98.0	
Elhadi F. 2015^6^ Sudan	Refugee and IDP camps	210 internally–displaced children	75.2 (68.8–80.9)											
**Poliomyelitis vaccine (OPV, IPV)**														
Rossi R. 2016^24^ Lebanon	Households and collective shelters	210 Lebanese and refugee children aged 12–59 months	90.4 (84.0–96.7)/98.9 (96.8–99.9)^i^	81.9 (73.6–90.2)/95.6 (91.5–99.8)^i^	65.1 (54.8–75.3)/92.4 (87.0–97.8)^i^	38.8 (27.1–50.5)/85.7 (78.2–93.2)^i^	43.4 (32.7–54.0)/85.7 (78.8–93.0)		92.9 (88.5–97.4)/97.5 (94.6–99.9)^i^	85.8 (79.8–91.9)/94.1 (91.0–98.9)^i^	76.4 (69.0–83.8)/88.1 (82.3–94.0)^i^	54.5 (44.7–64.2)/80.0 (72.0–88.0)^i^	57.5 (48.9–66.1)/83.1 (76.3–89.8)^i^	
Mansour Z. 2019^8^ Lebanon	Households	9315 children 7136 Lebanese 2179 Syrian refugees	94.5 (92.2–96.2)	84.5 (81.3–87.2)	76.5% (73.1–79.6)^a^				96.1 (95.2–96.8)	91.5 (90.3–92.6)	87.4 (86.0–88.7)^a^			
Alawieh A. 2017^11^ Lebanon	Surveillance system	5,882,562 Lebanese and 1,890, 840 Refugees					Adults 95.0 Children Syrian 32.5 Palestinian 63.0	0 47.5					91.0 95.0	0.0 0.0
Farag N. 2020^22^ Jordan	Households in High–Risk Areas (HRAs) and refugee camps	HRAs: 375 Jordanian and 104 refugee children Camps: 276 Syrian refugees			OPV 81 (76–86) IPV 75 (68–81)			99 (96.8–99.7)						
**Diphtheria, Tetanus and Pertussis containing vaccines (DTP, DTwP) and Pentavalent**														
Mansour Z. 2019^8^ Lebanon	Households	7136 Lebanese and 2179 Syrian refugee children	89.4 (86.7–91.6)	83.7 (80.8–86.2)	76.6 (73.5–79.5)				95.9 (95.0–96.6)	92.5 (91.4–93.4)	88.8 (87.4–90.0)^b^			
Ministry of Health 2019^19^ Saudi Arabia	NR	88 Saudi and 6 Non–Saudi children	81.8 (35.9–99.6)	75.2 (35.9–99.6)	59.7 (22.2–95.6)	23.4 (0.42–64.1)			89.8 (83.5–96.1)	79.8 (71.4–88.1)	68.5 (58.9–78.2)	29.2 (19.7–38.8)		
Rossi R. 2016^10^ Lebanon	Households and collective shelters	210 Lebanese and refugee children aged 12–59 months	86.7 (79.5–94.0)/97.8 (94.8–99.9)^i^	81.9 (73.6–90.2)/95.7 (91.5–99.8)^i^	65.1 (54.8–75.3)/89.1 (82.8–95.5)^i^	38.8 (27.1–50.5)/69.0 (59.2–78.9)^i^	43.4 (32.7–54.0)/69.6 (60.2–79.0)^i^		89.0 (83.6–94.4)/94.1 (89.8–98.3)^i^	80.3 (73.4–87.2)/86.4 (80.3–92.6)^i^	74.1 (66.4–81.6)/82.2 (75.3–89.1)^i^	47.5 (37.8–57.3)/71.6 (62.5–80.6)^i^	52.8 (44.1–61.4)/73.7 (65.8–81.7)^i^	
**Hepatitis B (HepB)**														
Mansour Z. 2019^8^ Lebanon	Households	7136 Lebanese and 2179 Syrian refugeechildren	67.0 (63.0–70.8)^h^	87.9 (85.1–90.2)	82.2 (79.1–84.9)	71.5 (68.0–74.8)			86.6 (84.8–88.2)^h^	95.2 (94.2–96.0)	91.9 (90.7–92.9)^c^	84.8 (83.1–86.2)		
Khan A. 2008^17^ Saudi Arabia	Community	665 blue collar expatriate workers					56.0 (52.1–80.9)							
**Rotavirus vaccine (RV)**														
Mansour Z. 2019^8^ Lebanon	Households	9315 children 7136 Lebanese 2179 Syrian refugees	69.2 (65.4–72.7)						72.4 (70.3–74.4)^d^					
**Meningococcal vaccine (MV)**														
Mansour Z. 2019^8^ Lebanon	Households	7136 Lebanese and 2179 Syrian refugee children	79.3 (76.1–82.1)	51.6 (48.1–55.1					86.7 (85.3–88.0)	64.8 (62.7–66.9)^e^				
**Oral Cholera Vaccine (OCV)**														
Lam E. 2017^7^ Iraq	Households in refugee and IDP camps	5007 refugees and IDPs	7.0 (6.0–9.0)	87.0 (85.0–89.0)										
***Haemophilus influenzae* b (Hib) vaccine**														
Mansour Z. 2019^8^ Lebanon	Households	7136 Lebanese and 2179 Syrian refugee children	88.4 (85.6–90.7)	83.4 (80.5–86.1)	76.0 (72.7–79.0)				95.3 (94.3–96.2)	92.3 (91.2–93.3)	88.7 (87.3–89.9)^f^			

a AOR = 0.6, 95% CI (0.5–0.7).

b AOR = 0.6, 95% CI (0.5–0.8).

c AOR = 0.7, 95% CI (0.5–0.8).

d AOR = 0.9, 95% CI (0.7–1.0).

e AOR = 0.7, 95% CI (0.6–0.8).

f AOR = 0.7, 95% CI (0.6–0.8).

g As per the definition used in this review.

h Birth dose.

i Study reported both pre–campaign/post–campaign data.

Across four studies on polio^38^^,^^41^, ^42^, ^43^ OPV1 and OPV3 coverage was 90.4–94.5% and 65.1–76.5% in migrants, respectively, compared to 92.9–96.1% and 76.4–87.5% in host children.^38^^,^^43^ Full-vaccination coverage for polio was 32.7–43.4% in migrants vs 54.4–87.4% in hosts.^38^^,^^41^ For DTP containing vaccines, coverage for DTP1 (indicator for zero-dose children) was 81.8%–86.7% in migrants vs 68.5%–88% in host children,^38^^,^^43^^,^^44^ whereas coverage for DTP3 (indicator for coverage) was 59.7%–76.6% in migrant children and 89.0%–95.9% in host children.^38^^,^^43^^,^^44^

Overall, migrant children showed a higher drop-out rate between the first and latest dose in the primary series across six vaccines (MCV, OPV, DTP containing vaccines, HepB, MV, and Hib vaccine) in two studies.^38^^,^^43^ Amongst 2262 migrant children, the lowest drop-out was 12.4% and the highest was 38.5% whereas in 7263 host children, drop-out was 6.6%–31.5% respectively. The highest dropout rates recorded were in OPV, MCV, and DTP containing vaccines (see Table 3).^38^^,^^43^ Finally, one study found that migrant children have significantly lower odds of receiving the 3rd doses of OPV, HepB, the 2nd dose of Meningococcal vaccine, and the first dose of RV compared to host children (see Table 3 for ORs).^43^

### Adult vaccination coverage

Nine studies reported on the coverage for any dose of the COVID-19 vaccine.^48^, ^49^, ^50^, ^51^^,^^61^ Coverage for at least one dose of COVID-19 vaccine was 33.5–84.8% in migrants (8 studies) compared to 25.0–59.0% in host populations (5 studies) (see Table S4—Supplementary Material). One study assessed distribution of vaccinated healthcare workers across nationality, finding that being migrant was associated with higher odds of receiving the vaccine (185/736 migrants vs 167/322 host, AOR = 1.918 (95% CI 1.36–2.69).^59^ The two remaining studies on adult vaccination reported coverage of all doses of hepatitis B and polio vaccines, finding low coverage for HepB (56%) and high coverage for OPV (95%) (see Table 3).^41^^,^^46^

### Vaccine acceptance and hesitancy

Eight studies—all on COVID-19—reported on vaccine acceptance or hesitancy.^40^^,^^49^^,^^55^^,^^60^, ^61^, ^62^, ^63^, ^64^, ^65^ In three studies, COVID-19 vaccine acceptance ranged from 61.0% to 89.6% among 3521 migrants in Jordan and Lebanon.^55^^,^^60^^,^^61^ Socio-demographic predictors of high vaccine uptake in migrants were reported in one study and included younger age, being female, having higher education and living inside informal tent (see Table S3 in Supplementary Material for OR).^61^ With respect to hesitancy, a study in Jordan found that being a migrant was associated with lower odds of COVID-19 vaccine hesitancy compared to host populations (49.9% in 501 migrants vs 60.0% in 491 nationals (OR 1.50, 95% CI 1.17–1.93).^49^

### Vaccination policies

Twenty-five studies included information on vaccination policies^66^, ^67^, ^68^, ^69^, ^70^, ^71^, ^72^, ^73^, ^74^, ^75^, ^76^, ^77^, ^78^, ^79^, ^80^, ^81^, ^82^, ^83^, ^84^, ^85^, ^86^, ^87^, ^88^, ^89^, ^90^, ^91^ of which 10 studies were in GCC countries,^67^^,^^71^^,^^72^^,^^75^^,^^79^^,^^80^^,^^84^^,^^87^^,^^89^^,^^90^ and 15 studies were on COVID-19.^66^, ^67^, ^68^, ^69^, ^70^, ^71^, ^72^, ^73^^,^^83^^,^^84^^,^^86^^,^^87^^,^^89^^,^^90^

For COVID-19 vaccination, all migrants (regular, undocumented, IDPs and refugees) were included in national roll-out plans in all 16 MENA countries.^26^ However, an IOM report showed that only six out of 16 countries included undocumented migrants in practice.^26^ Saudi Arabia, Lebanon, Bahrain, Tunisia, and Morocco also included undocumented migrants in COVID-19 national vaccination campaigns.^66^, ^67^, ^68^, ^69^, ^70^, ^71^, ^72^, ^73^^,^^83^^,^^84^

For childhood vaccination, six countries (Egypt, Jordan, Palestine, Tunisia, Algeria and Morocco) provide free of charge vaccination to newly arrived migrant children and children of migrants, regardless of legal status.^75^, ^76^, ^77^ Lebanon also provides free vaccinations to all, including refugees,^85^^,^^88^ and in response to the Syrian refugee crisis, the country set-up vaccination points at borders and registration centres for early vaccine access, alongside robust targeted polio and measles campaigns.^85^^,^^88^^,^^91^ Bahrain also provides free of charge measles vaccination to non-Bahraini resident children.^75^ For adolescents, Jordan and Tunisia provide the polio vaccine and MCV to all newly arrived adolescents, while Egypt offers the polio vaccine to adolescents from high-risk countries.^77^

For adults, GCC countries have a mandatory requirement for vaccination against Polio, MMR1 and 2, and Meningococcal vaccine for all for adults seeking to work or reside in these countries.^79^^,^^80^ Jordan provides the tetanus vaccine to newly arrived child-bearing age mothers^77^ and polio vaccine to adults coming from high-risk countries^77^ (see Table S5 in Supplementary Material—description of policies for further details).

### Behavioral and social drivers of vaccination uptake

**Fig. 3:**
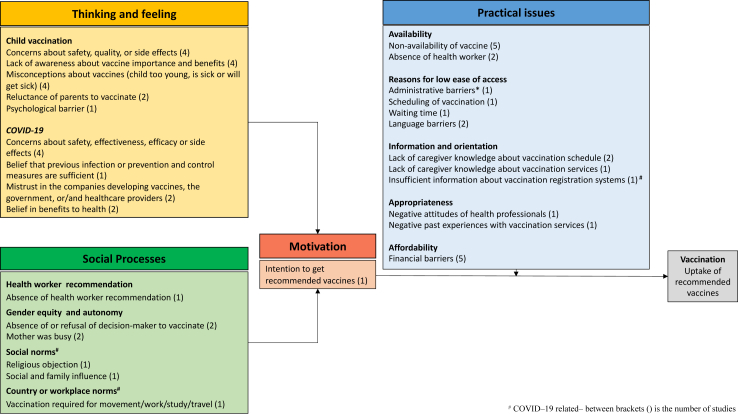
**Behavioral and social drivers of vaccination uptake among migrants in the MENA region**.

A total of 14 studies included information on drivers of uptake^40^^,^^42^^,^^43^^,^^45^^,^^47^^,^^49^^,^^56^, ^57^, ^58^^,^^60^, ^61^, ^62^^,^^92^^,^^93^ including six on childhood vaccination^40^^,^^42^^,^^43^^,^^45^^,^^47^^,^^93^ and eight on COVID-19 in adults,^49^^,^^56^, ^57^, ^58^^,^^60^, ^61^, ^62^^,^^92^ reflected in the WHO BeSD framework (Fig. 3).

For childhood vaccination, the main drivers of vaccine uptake were related to practical accessibility issues. These included vaccine shortages,^40^^,^^42^^,^^43^^,^^45^^,^^93^ the unavailability of healthcare workers or vaccination staff,^40^^,^^45^ language difficulties,^47^^,^^93^ financial concerns,^43^^,^^47^^,^^93^ administrative issues,^47^ waiting time,^47^ and lack of information regarding vaccination schedules and services (Fig. 3).^47^^,^^93^ Six studies reported drivers related to thoughts and feelings about childhood vaccines among caregivers.^40^^,^^42^^,^^43^^,^^45^^,^^47^^,^^93^ These included concerns about vaccine safety, quality or side effects^40^^,^^43^^,^^45^^,^^93^ reluctance of parents or caregivers to vaccinate,^43^^,^^93^ and lack of caregiver knowledge about the importance and benefits of vaccination.^40^^,^^42^^,^^45^^,^^47^ Social processes underlying uptake were reported in two studies and included the lack of autonomy and decision-making power by mothers regarding child vaccination, household and professional responsibilities of the mother,^40^^,^^45^^,^^93^ and absence of health worker recommendation.^43^

Drivers of uptake for adult vaccination were all focused on COVID-19.^49^^,^^60^, ^61^, ^62^, ^63^^,^^92^ These were mainly related to the thoughts and feelings domain, including concerns about effectiveness, safety, quality, and side-effects,^49^^,^^56^^,^^60^^,^^62^ beliefs that previous infection or safety measures are sufficient,^49^ and mistrust of healthcare workers, hospitals and companies developing the vaccines.^49^^,^^58^ Social processes driving COVID-19 vaccine uptake included workplace norms (being able to travel and move, or country/institution requirement), religious objections,^92^ and social and family influence.^56^ With respect to motivations, one study reported intention to getting the COVID-19 vaccine as a predictor of uptake of at least one dose of the vaccine.^61^ Only two studies reported on practical accessibility issues for COVID-19 vaccination which included the lack of information and orientation related to vaccination and registration systems,^49^ and financial barriers.^57^

## Discussion

In this systematic review and meta-analysis, we have reported vaccination coverage, acceptance, policies, and drivers of uptake among migrants in the MENA region. We found that the evidence base on vaccination was sparse, with most studies focusing on COVID-19, limited studies on adult and catch-up vaccination, few studies comparing migrant to host populations within countries, and very limited data in North Africa. Nevertheless, we found that in some countries across the region, migrants were entitled to free childhood vaccinations yet despite this entitlement, rates of childhood vaccination in migrants were low. Only 36.0% (95% CI 35.0–43.0%) of migrant children in this review completed vaccinations according to national schedules, which is far below national coverage estimates reported in some MENA countries.^94^^,^^95^ Likewise, we found a generally low coverage for key individual childhood vaccines in migrants, (e.g. DTP1 81.8%–86.7%; DTP3 59.7%–76.6%); OPV3 65.1%–76.4%; MCV1 63.9%–66.9%; MCV2 25.4%–85.6%); and high drop-out rates for subsequent doses (12.4%–38.5%), exceeding the 10% cut-off point set by the WHO.^96^ We identified key drivers of childhood vaccination uptake, including limited vaccine availability, staffing shortages, communication and administrative issues, financial constraints, and caregiver distrust. Overall, COVID-19 vaccines were provided free to migrants, but uptake was largely hindered by distrust in the vaccine and healthcare systems. Our data suggests that tailored interventions are urgently needed to drive uptake and coverage across all vaccine doses for both children and adult migrants, with a renewed focus on adopting a life-course approach and also targeting specific groups which have not been adequately studied such as migrant workers.

Recognising the limited evidence base, our results of low vaccination coverage in children align with those from other regions of the world showing that migrant children are an under-vaccinated group.^97^ Globally, a recent meta-analysis among migrants in EU/EEA countries also found that protective immunity against measles and diphtheria among children was below herd immunity thresholds.^98^ Similarly, a global meta-analysis of 7,375,184 participants in more than 10 countries also found that, regardless of age, migrants were half as likely to be vaccinated compared to non-migrants.^7^ In this review, we found a vaccine completion rate of 36% (95% CI 35–43%) among migrant children, which is much lower than the national estimates for fully vaccinated children in host populations across the MENA region. For example, the rates are 90.6% in Morocco, 61% in Algeria, and 77.8% in Jordan based on the latest data.^94^^,^^95^ In contrast, the gap is narrower when comparing this figure to that of fully vaccinated children in countries which have experienced protracted conflicts, such as Yemen (29.3%) and Iraq (41.6%).^95^ Similarly, coverage for DTP3, which is a global indicator for vaccine coverage, was around 59.7%–76.6% in migrants in this review, while across the MENA region, the rate is similar in Lebanon (63.21%), and Sudan (84%) but higher in countries such as Morocco (99.0%) and Kuwait (99%).^19^ It is important to acknowledge that national vaccination coverage, even in non-migrant populations, has dropped markedly in war-torn MENA countries since the conflicts began and has stagnated ever since^99^ with multiple polio and measles outbreaks disproportionately affecting children in conflicts and displaced settings. This situation has also been exacerbated by the backslide in immunisation experienced due to COVID-19 where coverage for DTP3 and MCV dropped by 1–2% in the region.^100^

In the MENA region, international organizations address the vaccination needs of migrants and vulnerable host communities through robust SIAs and healthcare system strengthening. They also advocate for migrant inclusion in national vaccination policies. However, despite these efforts, gaps persist between migrants' policy entitlements and their low actual vaccination coverage, likely due to persistent barriers identified in our review. The most common drivers for vaccination in migrant children in the MENA region were related to practical accessibility issues such as non-availability of the vaccine, absence of the healthcare workers, insufficient information and orientation, as well as those related to the role of caregiver knowledge, beliefs and awareness. These findings are consistent with another systematic review showing that migrants globally experienced practical accessibility issues such as communication and language challenges, administrative and legal issues and a lack of knowledge on awareness of need for, and availability of vaccination.^8^ Similarly, in the Eastern Mediterranean Region, reports show that the lack of awareness and insufficient knowledge about MCV and polio prevented timely vaccination among migrants, hindering polio eradication efforts.^101^ Few MENA countries in this review extended their vaccination offer to adolescents and adults (other than COVID-19).^76^ Nevertheless, the WHO calls for the strengthening of global immunisation throughout the life course by countries adapting their vaccination policies to include migrants in routine services, and by building inclusive and robust information systems, improving vaccine literacy, and addressing both informal and structural barriers to vaccination uptake.^102^ Our findings suggest that routine vaccination is complex and efforts to increase uptake should address both health system related barriers such as shortages in health staff and inadequate supply of vaccine, which affect both migrants and nationals, as well as entitlement and raising awareness about vaccination to migrant parents and caregivers through targeted outreach interventions. Panel 1 outlines key areas for policy, practice, and research going forward.

Panel 1 **Key policy, practice and research implications**

**Policy**

• Ensure legal access to vaccination services for migrants of all ages regardless of immigration status for routine immunisation including occupational vaccines (i.e. migrant workers) and newly introduced vaccines.
• Systematically integrate migrants into routine health information systems to capture coverage, uptake and completion of recommended vaccines.
• Collaborate with implementing partners such as non-governmental organisations (NGOs), civil society organisations (CSOs), and humanitarian actors in the MENA region to address persisting administrative barriers to accessing vaccination services and to increase knowledge among migrant communities about their entitlement to services.
• Develop national catch-up vaccination policies and provide adequate funding to health systems to introduce and deliver catch-up vaccination programmes.
• Increase data collection on vaccine acceptance and demand among migrants in the MENA region to build a stronger evidence base for policy and practice

**Practice**

• Provide training to health workers to raise awareness about life-course vaccination for migrant groups particularly those with uncertain or incomplete vaccination status.
• Develop tailored catch-up vaccination programmes to address vaccine incompletion and missed doses in newly arrived adolescent and adult migrants.
• Work with migrant communities to co-design tailored interventions, promote trust in vaccines and support vaccine champions to engage migrant communities in these initiatives.
• Identify alternative and innovative vaccine delivery models using lessons learned during the COVID-19 such as the deployment of outreach strategies, collaboration with faith-based and community-based organizations and flexible working hours, and leverage successful models in delivering health interventions in conflict zones.
• Systematically integrate vaccination services with other services offered to migrants upon arrival, in the community, camps, or other settings and across different access points.
• Collaborate with migrant communities and grass-root migrant community organisations to co-develop approaches to vaccination information which reach the migrant groups and address persisting concerns and rumours surrounding vaccination to promote trust in vaccines.

**Research**

• Conduct national-level robust data collection on uptake of routine vaccination (disaggregated by migrant status, country of origin, age and gender) to generate accurate coverage estimates across different groups, identify gaps and inequalities in uptake, and monitor unmet vaccination needs in migrant groups.
• Invest in large scale research to better understand the drivers of under-vaccination and vaccine hesitancy among different migrant groups and identify the most effective interventions to address drivers of non-uptake and the appropriate access and intervention points.
• Conduct studies to explore the most used and trusted information channels among specific groups to increase the reach of information on vaccination services and entitlement to free services.
• Support research among the most marginalised and under-studied migrant groups such as undocumented migrants, migrant workers and those at high-risk of VPDs, to identify specific barriers to accessing vaccination for these groups.

The existing evidence on vaccination coverage and drivers of vaccination uptake for migrant populations in the MENA region is of poor quality and is marked by significant gaps. The accuracy of vaccination status is dependent on the availability of high-quality records. Studies reporting on coverage in this review used both health card and recall methods to assess vaccination status, introducing potential recall and social desirability biases. The majority of studies were also small-scale, primarily relying on surveys among limited samples of participants. This may be driven by the unavailability of data, leading to studies being conducted solely among accessible populations. National state census and registry data, known for their accuracy,^103^ might offer more precise descriptions of coverage. Additionally, there is an absence of disaggregated coverage data, particularly by legal status, nationality, setting, time of stay in the country, and other socio-demographic factors. Thus, the current knowledge base on drivers of uptake also remains limited and fails to capture the diverse migrant groups living in the MENA region. Finally, there is a limited number of studies on adult vaccination with most of the literature focusing on COVID-19, and an absence of studies on adult catch-up vaccination. This marks a significant gap in the evidence base as some studies in Europe suggest that adults more likely to be under-vaccinated compared to children due to missed vaccines and vaccine doses as children.^104^^,^^105^

Given the limitations of the current evidence base, future studies should explore coverage levels for child and adult catch-up vaccination across different subgroups. Regional and national efforts to include migrants in routine health information systems are needed to identify low pockets of immunisation, track defaulters and address the immunisation needs of this group. In addition to better data, participatory approaches such as co-designing interventions with migrant communities,^106^ could be used to explore innovative vaccination delivery approaches to increase uptake within the MENA context. Most studies defined migrants as “non-nationals” or refugees, overlooking groups like irregular and low-skilled workers who face unique challenges, including deportation fears.^66^ Future research should better understand coverage for these vulnerable subgroups and the specific barriers they face. Finally, adult and catch-up vaccination services have not been sufficiently studied, and future research should consider examining the coverage, mapping barriers and vaccine delivery interventions, and surveying policies and practices related to adult and catch-up vaccination for migrants in the MENA region.

This review, despite being very large, has some limitations. Excluding studies before 2000 may have led to the omission of high-quality literature published earlier. However, this decision was made to ensure that the review is relevant to recent migration patterns and infections and we believe that earlier literature would not alter our recommendations. In addition, the definition of the MENA region used may have excluded certain countries included in broader definitions by other organisations. While we have performed extensive grey literature searches and conducted expert checks, we did not hear back from several experts. As a result, our search remains limited to what is available in the published literature. Understanding the realities of vaccination for migrants in the region ultimately requires direct engagement with stakeholders in each country, examining available national databases, and carrying out field assessments. Moreover, the limited number of studies prevented robust meta-analyses and meta-regression analyses to identify the sources of heterogeneity between studies – which was moderately high in this meta-analysis. It is also important to acknowledge that factors such as living and working conditions, the availability of international aid, and legal issues impact vaccination access and coverage. Since the data in the included studies did not stratify by these factors, this remains a limitation of the review and limits our understanding of the differential levels of coverage and access. Finally, the scope of the results is constrained by the availability and quality of published evidence, which lacks large-scale coverage estimates with disaggregated data. This is a frequent issue in migrant health research due to historically inadequate data collection, the absence of large-scale studies, and the non-systematic inclusion of migrants in routine health information systems.

In conclusion, this review has shown that despite migrant inclusive policies for vaccination in many countries across the MENA region, migrants experience a range of practical and individual level drivers affecting uptake of vaccination services. The low percentage of fully vaccinated migrant children and the inadequate coverage for OPV, MCV, and DTP-containing vaccines suggest that migrants in the MENA region may be an at-risk group for VPDs. Therefore, they should be prioritised in vaccination outreach efforts to achieve the recommended HIT and reach disease eradication and elimination goals. Progress towards WHO IA2030 targets relies on strengthening efforts to include migrants and displaced people, to reduce inequality in coverage and increase opportunities for all children to complete all vaccines in the national schedules. It is therefore crucial for health policies in the region to adopt a life-course approach and deploy innovative strategies by working with communities on innovative and tailored delivery approaches to address the identified drivers.

## Contributors

FS, OB, AD, ARM, SH generated the protocol with input from all authors. OB and FS did the database search, OB and HEM did abstract, full-text screening, and data extraction. OB and FS did the data analysis. OB and FS wrote the first draft of the manuscript with input from all the authors. SH supervised the work. SH and OB accessed and verified the data.

## Data sharing statement

Data are available on reasonable request from the corresponding author.

## Declaration of interests

The authors declare no competing interests.

## Appendix A Supplementary data

Supplementary data

Translated Abstracts
