# Supplementary material for: Vaccination coverage and access among children and adult migrants and refugees in the Middle East and North African region: a systematic review and meta-analysis
Source: eClinicalMedicine. 2024 Nov 22;78:102950. doi: 10.1016/j.eclinm.2024.102950 (PMC11647140; doi:10.1016/j.eclinm.2024.102950)
Supplement: Supplementary data [file mmc1.docx]

**Vaccination coverage and access among children and adult migrants and refugees in the Middle East and North African region: A systematic review and meta-analysis**

**Supplementary material**

Oumnia Bouaddi, Farah Seedat, Hassan Edries Hasaan Mohamad, Stella Evangelidou, Anna Deal, Ana Requena-Méndez, Mohamed Khalis, Sally Hargreaves on behalf of the Middle East and North Africa Migrant Health Working Group

**Methods**

**Table 1. Search strategy**

**Medline**

| Interface: Ovid MEDLINE(R) and Epub Ahead of Print, In-Process & Other Non-Indexed Citations and Daily  Date of Search: 27 August 2024  Number of hits: 1565  Comment: In Ovid, two or more words are automatically searched as phrases; i.e. no quotation marks are needed | Field labels   - exp/ = exploded MeSH term - / = non exploded MeSH term - .ti,ab,kf. = title, abstract and author keywords - adjx = within x words, regardless of order - * = truncation of word for alternate endings |
| --- | --- |
| Database(s): **Ovid MEDLINE(R) ALL**1946 to August 26, 2024 Search Strategy:   \| **#** \| **Searches** \| **Results** \| \| --- \| --- \| --- \| \| 1 \| exp Human Migration/ \| 28201 \| \| 2 \| exp "Emigrants and Immigrants"/ \| 16379 \| \| 3 \| "Transients and Migrants"/ \| 14976 \| \| 4 \| Refugees/ \| 14046 \| \| 5 \| Refugee Camps/ \| 344 \| \| 6 \| (alien* or asile or asylum* or (border* adj2 cross*) or (countr* adj3 origin*) or diaspora or displace? or displacement* or emigrant* or emigration or expat? or expatriate? or foreigner* or foreign-born* or foreign background* or foreign population* or immigrant* or immigration or migrant* or migration or naturalized citizen* or new* arriv* or newcomer* or new-comer* or nomad* or non-citizen* or nonnative* or non-native* or nonnational or non-national or nonresident or non-resident* or resettlement* or re-settlement* or refugee* or settler* or squatter* or undocumented worker*).ti,ab,kf. \| 629435 \| \| 7 \| or/1-6 \| 643313 \| \| 8 \| exp Middle East/ \| 170231 \| \| 9 \| exp Africa, Northern/ \| 43213 \| \| 10 \| (Abu Dhabi or Ajman or Algeri* or Arab* or Bahrain* or Bahreiin* or Dubai or Egypt* or Emirat* or Fujairah or Gaza* or Golf* or Gulf* or Ifriqiya* or Irak* or Iraq* or Jorda* or Jumhuuriiya* or Koweit* or Kuwait* or Kuwayt* or Leban* or Liban* or Liby* or Lubnan* or Maghr* or Maroc* or Maser* or Masr or Misr or MENA or Middle East* or Morocc* or North* Afric* or Oman* or Palestin* or Qatar* or Saudi* or Sharjah or Soudan* or Sudan* or Syria* or Syuri* or Tunis* or Uman* or Umm Al-Quwain or West Bank or Yemen*).ti,ab,kf. \| 353345 \| \| 11 \| or/8-10 \| 476281 \| \| 12 \| exp Vaccines/ \| 290640 \| \| 13 \| exp Immunization/ \| 218272 \| \| 14 \| exp Immunization Programs/ \| 16548 \| \| 15 \| (immunostimulation* or immunisation* or immunization* or vaccin* or variolation*).ti,ab,kf. \| 509141 \| \| 16 \| (immunologic* adj2 (sensiti?ation* or stimulation*)).ti,ab,kf. \| 619 \| \| 17 \| Vaccine-Preventable Diseases/ \| 273 \| \| 18 \| Cholera/ \| 9521 \| \| 19 \| COVID-19/ \| 273160 \| \| 20 \| exp Dengue/ \| 16940 \| \| 21 \| Diphtheria/ \| 7061 \| \| 22 \| exp Hepatitis B/ \| 66526 \| \| 23 \| Haemophilus influenzae type b/ or Haemophilus Infections/ \| 8849 \| \| 24 \| exp Papillomaviridae/ \| 39772 \| \| 25 \| Influenza, Human/ \| 60072 \| \| 26 \| Measles/ \| 15147 \| \| 27 \| exp Meningococcal Infections/ \| 11943 \| \| 28 \| Mumps/ \| 4968 \| \| 29 \| Whooping Cough/ \| 9413 \| \| 30 \| exp Pneumococcal Infections/ \| 22934 \| \| 31 \| exp Poliomyelitis/ \| 20859 \| \| 32 \| Rabies/ \| 11168 \| \| 33 \| Rubella/ \| 8450 \| \| 34 \| Rotavirus Infections/ \| 8907 \| \| 35 \| Tetanus/ \| 9937 \| \| 36 \| exp Tuberculosis/ \| 210398 \| \| 37 \| exp Varicella Zoster Virus Infection/ \| 20502 \| \| 38 \| (vaccine preventable adj3 (disease* or illness* or infection*)).ti,ab,kf. \| 4917 \| \| 39 \| (2019 ncov or 2019ncov or breakbone fever or break-bone fever or chickenpox* or chicken pox* or cholera* or cholerae or coqueluche or coronavirus 2 or corona virus disease 2019 or cov2 or cov 2 or covid-19 or covid19 or dengue* or diphtheri* or diphteri* or epidemic parotiti* or german measles or grippe or h1n1 or hav or haemophilus or hbv or hepa or hepatit* or herpesvirus 3 or hepb or hib or hpv or human flu or human papilloma* virus* or hydrophobia or influenza* or koch* disease or lyssa or lyssas or measles or meningit* or meningoco* or mumps or ncov or neisseria meningitidis or neonatal calf diarrhea virus* or new corona virus* or new coronavirus* or novel corona virus* or novel coronavirus* or oreillons or pachymeningiti* or papillomavir* or pertuss* or pfeiffer* bacillus or pneumococcal or pneumoniae or polio* or rabies or rotavirus* or rougeol* or rubella* or rubeol* or sars-cov-2 or sars2 or tdap or tdp or tetani or tetanus* or three day measle* or tuberculos* or varicella or whooping cough*).ti,ab,kf. \| 1491528 \| \| 40 \| or/12-39 \| 1917337 \| \| 41 \| 7 and 11 and 40 \| 1888 \| \| 42 \| (animals not humans).sh. \| 5218162 \| \| 43 \| 41 not 42 \| 1840 \| \| 44 \| limit 43 to yr="2000 -Current" \| 1565 \| | |

**Results**

**Table 2: Odds of being fully-vaccinated in migrant versus host children**

| **Author, year**  **Country** | **Study design** | **Study setting** | **Study population** | **AOR 95% CI** | **Factors adjusted for** |
| --- | --- | --- | --- | --- | --- |
| Kmeid M. 2019 ^1^  Lebanon | Cross–sectional | Nurseries, schools, summercamps, waiting rooms of paediatric clinics, and dispensaries | 83 Syrian refugees  488 Lebanese | 1.15 (95% CI 0.54-2.47 | Parents’ age and education, mother’s profession, place of residence, number of children, place of vaccination and medical counseling, having a regular pediatrician and insurance coverage |
| Rossi R. 2016 ^2^  Lebanon | Cross sectional | Households (including collective shelters) | Lebanese 127/118 (1st – 2nd survey)  Syrian refugees 83/92 | 0.89 (95% CI 0.20-3.94) | Sex, age, place of residence and having heard of the vaccination campaign |

**Table 3. Socio-demographic predictors of vaccine uptake**

| **Author (year)** | **Study setting** | **Study population** | **Type of vaccination** | **Socio-demographic factors** |
| --- | --- | --- | --- | --- |
| Toraimbe S. 2021 ^3^  Morocco | Primary care centers | 402 Sub-saharan African migrant mother-child dyads | Child vaccination (all recommended vaccine in the NIP) | Mother's education OR 4.89 (95% CI 1,91-12,56)  Professional status OR 0.41(95% CI 0,21-0,82) |
| Zeid B. 2022^4^  Lebanon | Households | 2906 Syrian refugees  1822 outside informal settlements  1084 inside formal settlements | COVID-19 | Living inside informal tent settlements compared to living outside 1.44 (1.24-1.66), p<0.001 and having higher education 1.23 (1.03-1.48), associated with higher uptake of at least one dose of the COVID-19 vaccine. |
| Nizam A. 2022^5^  UAE | Web-based, Universites | University students  385 Overall  33 UAE National  352 Expatriates | COVID-19 | Older age OR 0.97; 95% CI 0.96 –0.98 and being male OR 0.56–0.62; 95% CI 0.43–0.73 were significantly associated with lower odds of actual and perceived vaccine acceptance |
| AlAwadhi E. 2021^6^  Kuwait | Online via social media platforms | Citizens and non-  citizens  Kuwaiti 5651  Non-Kuwaiti 1,590 | COVID-19 | Older age was significantly associated with lower odds of perceived vaccine acceptance OR 0.37–0.61; 95% CI 0.26–0.95 |

**Table 4. COVID-19 vaccine coverage**

| **Author, year**  **Country** | **Study design** | **Study setting** | **Sample size** | **Migrant N (%)** | **Host N (%)** |
| --- | --- | --- | --- | --- | --- |
| Al–Hatamleh 2022 ^7^  Jordan | Cross–sectional | Refugee camp | 501 Refugees  491 Citizens | Refugees 328 (42.7)  Doses:  1 dose 41 (73.2)  2 doses 287 (40.3) | Jordanians 441 (57.3)  Doses:  1 dose 15 (26.8)  2 doses 426 (59.7) |
| Zeid B. 2022 ^4^  Lebanon | Cross sectional | Households | 2906 Syrian refugees | Two or more doses 806 (27.7) |  |
| Shehab M. 2021 ^8^  Kuwait | Cross–sectional | University ospital | 201 Citizens and  79 Expatriates with IBD | Any dose 67 (84.8) | 50 (25.0) |
| UNHCR 2022^9^  Jordan | Monthly report | Camps and nationwide | Syrian refugees | Any dose 40.2 | 45.7 |
| UNHCR 2022 ^10^  Lebanon | Monthly report | Camps and nationwide | Syrian refugees | Any dose 33.5 | 59.0 |
| Ali M. 2024 ^11^  Jordan | Cross-sectional | Primary healthcare centres, schools, public areas and lounges | 259 Palestinians  200 Syrians  93 Non-arabs  84 Arabs | 370 (58.2) |  |
| Gubari M. 2023 ^12^  Iraq | Cross-sectional | Households | 3519 from host communities  428 IDPs  617 refugees | 1 dose  IDPs: 48.83  Refugees : 45.9  2 doses  35.7  36.1  3 doses  0.93  1.5 | 51.5  42.3  1.65 |
| Dalky A. 2024 ^13^  Jordan | Cross-sectional | Two refugee camps | 385 Syrian refugee women | 1 dose 16 (4.20)  2 dose 346 (89.9) |  |
| Aschore M. 2024 ^14^  Libya | Cross-sectional | Households | 1448 migrants  2019 refugees | Any dose 1,643 (47.6) |  |

**Table 5. Description of policies**

**a. COVID-19 vaccines**

| **Author (year)** | **Country** | **Publication type** | **Target population** | **Description of Policy** |
| --- | --- | --- | --- | --- |
| Tazyeen S. 2022^15^  Middle East | Middle East | Perspective | Foreign workers with a valid emirates ID | Provision of the COVID-19 vaccination to foreign workers with valid Emirati ID but in UAE could specifically not provide for undocumented workers due the valid ID being a compulsory requirement |
| Rahman M. 2022  Gulf countries | Gulf countries | Narrative review | Migrant workers | Employers are under obligation to cover their foreign employees’ health insurance and monitor workers' health status including vaccination. |
| Assiri A. 2021^16^  Saudi Arabia | Saudi Arabia | Commentary | Expatriates | Provision of free of charge COVID-19 vaccine to all Saudi citizens and residents including illegal residents |
| Habersky E. 2021^17^  EMR | EMR | Narrative review | Refugees registered with UNHCR | Refugees registered with UNHCR and migrants are allowed to register for the vaccine (AstraZeneca or Sinopharm), via the online vaccine registration portal of the MoH. |
| Ministry of Health 2021^18^  Tunisia | Tunisia | Strategy document | Migrants, refugees and individuals with irregular status | All migrants including refugees and irregular migrants are included in the national free of charge COVID-19 vaccination campaign with the exception of ineligible individuals such as under 18 and pregnant women. |
| Suliman D. 2021^19^  UAE | UAE | Commentary | Citizens and residents | Provision of free of cost COVID-19 vaccines to citizens and residents alike |
| UN Network for Migration 2021 ^20^  Bahrain | Bahrain | Policy brief | Citizens, residents and undocumented migrants | Collaboration of the National Task Force with embassies (India, Philippines and Bangladesh) to reach out to and register undocumented migrants seeking COVID-19 vaccines, through the organization of community-based vaccination days in a mall. Vaccination certificates were issued.  Barriers:  -undocumented migrants hard to reach and reluctant to come forward,  -domestic workers harder to reach & more vulnerable compared to commercial workers |
| WHO 2022^21^  Bahrain | Bahrain | Case study | Residents, regardless of nationality, residential status, or  Ethnicity | Provision of vaccinations free of charge to all Bahraini citizens and residents, regardless of nationality, residential status, or ethnicity, based on a direction from His Majesty the King of Bahrain |
| Ministry of Public Health 2021 ^22^  Lebanon | Lebanon | Guidelines | All residents regardless of nationality | All those residing in Lebanon and qualify for vaccination (above 16 years) will be provided free of charge COVID-19 vaccine (Pfizer) irrespective of nationality through the COVAX facility |
| IOM 2021^23^  Egypt | Egypt | Report | Migrants and refugees | MoPH launched the National Plan for COVID-19 Vaccination in January 2021. The Plan aims to vaccinate every citizen residing in Egypt, including migrants and refugees, through a nationwide campaign for registration online. |
| Rahman MM. 2023 ^24^ | Qatar | Narrative review | Migrant workers | Qatar residents, including low-skilled workers, received free doses of sophisticated vaccines like Pfizer-BioNTech, Oxford-AstraZeneca, Moderna, and Janssen, earlier than their counterparts in their home countries. |
| Chen S. 2024 ^25^ | Gulf countries | Narrative review | Residents | Vaccines provided free in the GCC countries for all citizens and residents. |
| Santus D. 2023 ^26^ | Morocco | Cross-sectional qualitative study | All migrants and refugees | Vaccination campaign migrants and refugees without identity papers. They can register in their neighborhood using a passport or proof of residence and create an account on the national COVID-19 vaccine registration platform. |
| Alahmad B. 2023 ^27^ | Gulf countries | Commentary | Non-nationals | In Kuwait, initial distribution of COVID-19 vaccine was limited to nationals (2021). |

**b. Other vaccines**

| **Author (year)** | **Country** | **Vaccines** | **Publication type** | **Target population** | **Description of policy** |
| --- | --- | --- | --- | --- | --- |
| Jawad J. 2011^28^  Bahrain | Bahrain | Measles | Cross sectional, descriptive | Citizens and residents | All measles-related activities, including both immunization and laboratory testing of suspected measles cases, are ﬁnancially supported by the government and provided free of charge in government health facilities for all Bahrainis and non-Bahraini residents. |
| Riccardo F. 2012^29^  North Africa | EpiSouth including North Africa (Algeria), Tunisia, Morocco) | NS | Cross-sectional study | Mobile communities (regular, irregular and nomadic) | Vaccines included in the NIP are provided and administered free of charge to children of migrants in vaccination centers. |
| Giambi C. 2017^30^  Mediterranean Basin and Black Sea | Mediterranean Basin and Black Sea (Jordan, Egypt, Tunisia, Palestine) | All routines vaccines | Cross-sectional study | Newly arrived migrants | -Immunization status is routinely verified by Palestine, Tunisia (for African student only) whereas Jordan does not verify it.  -Tunisia: all NIP vaccines are provided to children with particular attention to Libyan and Syrian foreign born. Polio, DTP and MMR also provided to adolescents.  -Palestine: all NIP vaccines provided to children at community level  -Jordan: All NIP vaccines provided to children especially Syrian refugees. For adolescents, measles vaccine is provided at age 11-15. Measles are provided at community level for 6 months–15years and poliomyelitis vaccine to children<5 years also inholding centres. For adults: tetanus is provided to child bearing women 15-49**.**  Egypt: All NIP vaccines provided to children less than 4 years old. Poliomyelitis vaccine is provided to children at any age and adolescents, and adults, coming from a country at polio risk (at entry level). Vaccinations are provided at community level. |
| Ministry of Health 2023^31^  Syria | Syria | Polio | Epi bulletin | IDPs and returnee migrants | Vaccination status of children incoming from Northeastern Syria, Raqa, Deir Zour, Idlib (conflict region) and returnees are provided polio vaccines |
| Gulf Health Council 2021^32^  Gulf countries | GCC | All routines vaccines | Regulations | Expatriates coming to GCC states for work or residence | All expatriates coming to reside or work in GCC states undergo a medical examination for fitness where vaccination status for (polio, MMR 1 & 2 Meningococcal and COVID-19) are assessed & vaccines provided by accredited centers |
| Gulf Health Council 2021^33^  Gulf countries | GCC | Poliomyelitis, MMR 1 and 2, meningococcal, COVID-19 (optional) | Technical guidelines | Expatriates coming to GCC states for work or residence | Any person seeking residency in GCC undergoes medical examination in an accredited center which; reviews vax record of candidate, determines the vaccines the applicant needs based on his or her age, records, documented  immunity, administers vaccines, in order to reside in GCC proofs of having vax or immunity required for Polio, Meningococcal MMR. Accepted vaccination records also include Laboratory evidence of immunity for MMR and polio |
| WHO 2018 ^34^  EMR | EMR | All | Report | All foreign-born | Provision of free vaccine to all patients visiting primary care facilities regardless of nationality, including subsidized services to displaces populations and refugees |
| IOM 2021^35^ | MENA | All routine vaccines | Report | Refugees and asylum seekers, regular and irregular migrants, IDPs | In the MENA region: 11/17 countries included refugees and asylum seekers in NVDP both in theory and practice, 15/17 included regular migrants in theory and 16 in practice, 11/17 included irregular migrants in theory but only 8 in practice, 4/7 included IDPs in theory and 3/7 in practice. |
| Ministry of Foreign Affairs 2017 ^36^ | Morocco | All routine vaccines | Policy evaluation | All migrants regardless of status | Free of charge to primary care services including vaccination for all migrants regardless of status |
| Alahmad B. 2023 ^27^ | Gulf countries | Flu and Pneumococcal vaccines | Commentary | Non-nationals | In Kuwait, initial restriction of flu and pneumococcal vaccines to Kuwaitis only (2020) |
| Honein-AbouHaidar G. 2024 ^37^ | Lebanon | All routine vaccines | Cross-sectional mixed-methods study | Syrian refugees | Provision of free immunizations through the MOPH's Expanded Program on Immunization, and through campaigns supported by UNICEF and WHO, ensures Syrian refugees' access to routine vaccines like polio and measles. Vaccination centres were also set up at the borders for Syrian children. |
| Ismail S. 2023 ^38^ | Lebanon | All routine vaccines | Cross-sectional qualitative study | Syrian refugees | Several adaptive changes took place in the Lebanese healthcare system in respnse to refugee arrival:  **Macrolevel:**  - National vaccination campaigns were launched in 2013-2014 with donor support to prevent outbreaks of polio and measles.  - Policy changes relaxed access rules, allowing displaced Syrians to receive vaccines at nominal costs, although hidden fees remained a barrier.  - Vaccination points established at border crossings and refugee registration centers to ensure early access to key vaccines.  **Mesolevel:**  - Mobile medical units (MMUs) were intensified to reach refugees in remote and rural areas where fixed clinics were limited, and to refer them to nearby PHCs.  - Community engagement activities scaled up to boost vaccination demand, provide education, and cross-check vaccination records, relying on increased recruitment of community workers and donor funding.  **Microlevel:**  - Task-shifting: redefining nurses' roles in vaccine administration and improving workforce skills to handle the increased demand from refugee populations. |

*MOPH: Ministry of Public Health*

**REFERENCES**

1 Kmeid M, Azouri H, Aaraj R, Bechara E, Antonios D. Vaccine coverage for Lebanese citizens and Syrian refugees in Lebanon. *Int Health* 2019; **11**: 568–79.

2 Rossi R, Assaad R, Rebeschini A, Hamadeh R. Vaccination Coverage Cluster Surveys in Middle Dreib - Akkar, Lebanon: Comparison of Vaccination Coverage in Children Aged 12-59 Months Pre- and Post-Vaccination Campaign. *PLoS ONE [Electronic Resource]* 2016; **11**: e0168145.

3 Esako Toirambe S, Camara T, Khalis M, *et al.* Facteurs prédictifs de la non-complétude vaccinale chez des enfants migrants de moins de 5 ans, Maroc. *Sante Publique (Paris)* 2021; **Vol. 33**: 435–43.

4 Zeid BA, Khoury T El, Ghattas H, *et al.* Predictors and barriers to vaccination among older Syrian refugees in Lebanon: a multi-wave survey. *medRxiv* 2022; : 2022.12.15.22282964.

5 Nizam A, Iqbal T, Mashood H, El Nebrisi E. Analyzing COVID-19 Vaccine Hesitancy among University Students in UAE: A Cross-Sectional Study. *Dubai Medical Journal* 2022; **5**: 182–93.

6 AlAwadhi E, Zein D, Mallallah F, Bin Haider N, Hossain A. Monitoring COVID-19 Vaccine Acceptance in Kuwait During the Pandemic: Results from a National Serial Study. *Risk Manag Healthc Policy* 2021; **14**: 1413–29.

7 Al-Hatamleh MAI, Hatmal MM, Mustafa SHF, *et al.* Experiences and perceptions of COVID-19 infection and vaccination among Palestinian refugees in Jerash camp and Jordanian citizens: a comparative cross-sectional study by face-to-face interviews. *Infect Dis Poverty* 2022; **11**: 123.

8 Shehab M, Zurba Y, Al Abdulsalam A, Alfadhli A, Elouali S. COVID-19 Vaccine Hesitancy among Patients with Inflammatory Bowel Disease Receiving Biologic Therapies in Kuwait: A Cross-Sectional Study. 2021; **10**: 31.

9 UNHCR. COVID-19 dashboard February 2022 - Jordan. 2022 https://reporting.unhcr.org/libraries/pdf.js/web/viewer.html?file=https%3A%2F%2Freporting.unhcr.org%2Fsites%2Fdefault%2Ffiles%2F2022-03%2FJordan%2520-%2520COVID-19%2520dashboard%2520-%2520February%25202022.pdf (accessed Jan 2, 2024).

10 UNHCR. Lebanon: COVID-19 response dashboard | Global Focus - Q3 2022. 2022 https://reporting.unhcr.org/lebanon-covid-19-response-dashboard (accessed Jan 2, 2024).

11 Ali M, Salam M. Factors associated with COVID-19 vaccine uptake and hesitancy among multinational refugees and migrants in Jordan. *International Migration* 2024; **62**: 57–81.

12 Gubari MIM, Wadi F, Hama-Ghareeb KA, *et al.* COVID-19 Vaccination Among Diverse Population Groups in the Northern Governorates of Iraq. *Int J Public Health* 2023; **68**: 1605736.

13 Dalky A, Quran TO, Abuhammad S, *et al.* COVID-19 vaccine acceptance and associated factors among pregnant and lactating women attending maternity care clinics in refugee camps in Jordan. *PLoS One* 2024; **19**. DOI:10.1371/JOURNAL.PONE.0305314.

14 Achore M. Correlates of COVID-19 vaccine uptake among the forcibly displaced: evidence from Libya. DOI:10.1186/s13690-024-01306-4.

15 Tazyeen S, Khan H, Babar MS, Lucero-Prisno 3rd DE. Foreign workers in the Middle East during COVID-19. *International Journal of Health Planning & Management* 2022; **37**: 1199–204.

16 Assiri A, Al-Tawfiq JA, Alkhalifa M, *et al.* Launching COVID-19 vaccination in Saudi Arabia: Lessons learned, and the way forward. *Travel Med Infect Dis* 2021; **43**: 102119.

17 Habersky E, Damir A. COVID-19 financing strategies for refugees and migrants in the Eastern Mediterranean Region. *Eastern Mediterranean Health Journal* 2021; **27**: 1229–38.

18 Redaction G DE. STRATEGIE VACCINALE CONTRE LA COVID-19 EN TUNISIE. .

19 Suliman DM, Nawaz FA, Mohanan P, *et al.* UAE efforts in promoting COVID-19 vaccination and building vaccine confidence. *Vaccine* 2021; **39**: 6341.

20 United Nations Network on Migration in Bahrain. 2021.

21 WHO Bahrain. BAHRAIN COVID-19 CASE STUDIES. https://www.moh.gov.bh/Content/Upload/File/637933076434107917-WHOEMBAH001E-eng.pdf (accessed March 2, 2023).

22 MOPH Lebanon. COVID-19 National Vaccination Campaign FAQs about COVID-19 Vaccine February 10, 2022. 2022. https://www.moph.gov.lb/userfiles/files/AwarenessCampaign/National%20Awareness%20Campaign%20on%20COVID%2019%20Vaccine%202021/COVID-19%20Vaccine%20FAQ%20-%20EN%20-%2010_02_2022.pdf (accessed Sept 28, 2023).

23 IOM Strategy for Egypt (2021–2025). 2020.

24 Rahman MM, Umar S, Almarri SA. Healthcare Provisions for Migrant Workers in Qatar. *Health Soc Care Community* 2023; **2023**: 6623948.

25 Chen S, Guo L, Xie Y, *et al.* Government responses to the COVID-19 pandemic of the Gulf Cooperation Council countries: good practices and lessons for future preparedness. *Glob Health Res Policy* 2024; **9**. DOI:10.1186/S41256-024-00349-Y.

26 Santus D, Ansaloni S, Santus D, Ansaloni S. Mobility issues and multidimensional inequalities: exploring the limits of the National Strategy for Immigration and Asylum during the COVID-19 pandemic in Morocco. *AIMS Geosciences 2023 1:191* 2023; **9**: 191–218.

27 Alahmad B, AlMekhled D, Busalacchi K, Wang WC. In-Depth Ethical Analysis of the COVID-19 Vaccine Rollout for Migrant Workers in the Gulf Countries. *International journal of social determinants of health and health services* 2023; **53**: 488–93.

28 Jawad JS, Al-Sayyad AS, Sataih F, Naouri B, Alexander Jr. JP. Toward measles elimination in Bahrain--a Middle East country experience. *Journal of Infectious Diseases* 2011; **204**: S299-304.

29 Riccardo F, Dente MG, Kojouharova M, *et al.* Migrant’s access to immunization in Mediterranean Countries. *Health Policy (New York)* 2012; **105**: 17–24.

30 Giambi C, Del Manso M, Dente MG, *et al.* Immunization Strategies Targeting Newly Arrived Migrants in Non-EU Countries of the Mediterranean Basin and Black Sea. *Int J Environ Res Public Health* 2017; **14**: 459.

31 Ministry of Health Syria. Summary of the current epidemiological situation of paralysis in Syria for the week 2023-32. .

32 Gulf Health Council. Regulations of Medical Examination of Expatriates Coming to GCC States for Work or Residence Sixth Version. 2021.

33 Gulf Health Council. Vaccination for Expatriates Coming to GCC States for Residence. .

34 Health of refugees and migrants Practices in addressing the health needs of refugees and migrants WHO Eastern Mediterranean Region 2018. .

35 International Organization for Migration (IOM). Migrant Inclusion in COVID-19 Vaccination Campaigns - IOM Country Office Review. 2021.

36 Ministry of  Foreign Affairs Morocco. Politique Nationale d’Immigration et d’Asile. 2017 www.marocainsdumonde.gov.ma (accessed Sept 17, 2022).

37 Honein-AbouHaidar G, Bou-Karroum L, Parkinson SE, *et al.* Integrating Syrian refugees into Lebanon’s healthcare system 2011–2022: a mixed-method study. *Confl Health* 2024; **18**: 1–20.

38 Ismail SA, Tomoaia-Cotisel A, Noubani A, *et al.* Resilience in childhood vaccination: analysing delivery system responses to shocks in Lebanon. *BMJ Glob Health* 2023; **8**: e012399.
