# Supplementary material for: Vaccination coverage and access among children and adult migrants and refugees in the Middle East and North African region: a systematic review and meta-analysis
Source: eClinicalMedicine. 2024 Nov 22;78:102950. doi: 10.1016/j.eclinm.2024.102950 (PMC11647140; doi:10.1016/j.eclinm.2024.102950)
Supplement: Translated Abstracts [file mmc2.docx]

The following translations in French and Arabic were submitted by the authors and we reproduce them as supplied. They have not been peer reviewed. Our editorial processes have only been applied to the original abstract in English, which should serve as reference for this manuscript.

**Couverture vaccinale et accès à la vaccination chez les enfants et les adultes migrants et réfugiés dans la région du Moyen-Orient et de l'Afrique du Nord : une revue systématique et méta-analyse**

Oumnia Bouaddi, Farah Seedat, Hassan Edries Hasaan Mohamad, Stella Evangelidou, Anna Deal, Ana Requena-Méndez, Mohamed Khalis, Sally Hargreaves

**Résumé**

**Contexte**
La région du Moyen-Orient et de l'Afrique du Nord (MENA) est un foyer majeur de migration mondiale, avec plus de 40 millions de migrants qui peuvent être sous-vaccinés en raison de barrières d’accès la vaccination dans les pays d'origine, de transit et de destination. Nous avons procédé à une synthèse systématique des données sur la couverture vaccinale, l'acceptation, les facteurs d’adoption et les politiques de vaccination pour les enfants et adultes migrants dans cette région, afin d'identifier des interventions adaptées pour cette population.

**Méthodes**
Nous avons consulté six bases de données (y compris Medline, Embase) pour la littérature évaluée par les pairs et des sites des organismes internationaux (tels que OIM, OMS, HCR) et des ministères de la santé dans la région) pour la littérature grise, couvrant la période de 2000 au 27 août 2024, dans toutes les langues, sur la couverture vaccinale, l’acceptation, les facteurs d’adoption et les politiques de vaccination pour les migrants dans la région MENA. Nous avons inclus les études présentant des données primaires sur la couverture vaccination et les facteurs d’adoption, ainsi que les articles pertinents sur les politiques de vaccination. Nous avons défini les migrants comme des personnes quittant leur lieu de résidence habituel, de manière temporaire ou permanente, au sein ou au-delà des frontières internationales. Les études sans données spécifiques aux migrants ont été exclues. Les résultats principaux étaient la couverture vaccinale (pourcentage d’individus ayant reçu au moins une dose de vaccin) et l’acceptation vaccinale (pourcentage d’individus acceptant tout type de vaccin). Nous avons analysé séparément les données pour les enfants (<18 ans) et les adultes (≥18 ans). Les estimations ont été regroupées via une méta-analyse à effets aléatoires ou synthétisées de manière narrative, et les facteurs d'adoption ont été analysés selon le cadre conceptuel Behavioral and Social Drivers of Vaccination (BeSD) développé par l’OMS. Protocole PROSPERO : CRD42023401694.

**Résultats**
En total, nous avons identifié 6,088 articles dans les bases de données et 282 rapports de littérature grise, et inclus 55 études portant sur 1 906 975 migrants dans 15 pays (principalement des réfugiés au Moyen-Orient et des expatriés dans les pays du Conseil de Coopération du Golfe). La vaccination contre la COVID-19 a été fournie gratuitement aux migrants dans tous les pays, tandis que la vaccination était accessible gratuitement pour les enfants migrants dans sept pays. Cependant, pour les adolescents et les adultes, nous avons relevé une grande variation entre les pays, et aucune politique de rattrapage vaccinal n’a été identifiée. La couverture vaccinale chez les enfants migrants était faible, avec seulement 36.0 % des 589 enfants migrants entièrement vaccinés selon les calendriers nationaux (IC à 95 % : 35.0 %-43,0 %, I2=67 % ; données provenant des migrants au Liban, au Maroc et au Soudan). Les données pour les vaccins de routine spécifiques aux enfants étaient également faibles : pour la rougeole, 63.9%-66.9 % pour la première dose et 25.4%-85.6 % pour la deuxième ; pour la poliomyélite, 65.1%-76.4 % pour la troisième dose ; pour la diphtérie, le tétanos et la coqueluche (DTP), 81.8 %-86.7 % pour la première dose et 59.7%-76.6 % pour la troisième. Les taux d'abandon pour des vaccins de routine variaient de 12,4 % à 38,5 %, indiquant que les migrants rencontrent divers obstacles au-delà de la première dose, nécessitant des interventions mieux adaptées. Pour les adultes, nous avons trouvé onze études sur la couverture vaccinale (dont neuf sur la COVID-19), montrant que la couverture contre la COVID-19 variait entre 33.5 % et 84.8 % chez les migrants et entre 25.0 % et 59.0 % dans les populations hôtes. Les facteurs de non-adoption de la vaccination infantile chez les migrants incluaient la disponibilité limitée des vaccins et du personnel, des obstacles administratifs et de communication, des difficultés financières, le manque de connaissance des services, et des préoccupations des parents concernant la sécurité et les bénéficies des vaccins. Chez les adultes, les facteurs de non-adoption étaient principalement liés au vaccin COVID-19 et incluaient des préoccupations sur la sécurité, la qualité, les effets secondaires, et une méfiance envers les vaccins et les systèmes de santé.

**Interprétation**
Les migrants présentent des facteurs de risque uniques de sous-immunisation dans la région MENA et ont une faible couverture vaccinale malgré un certain niveau de droit aux services. Les données sur la couverture vaccinale, les facteurs d'adoption et les politiques pour les migrants dans la région MENA sont limitées à des études de petite ampleur auprès de groupes accessibles, se concentrant principalement sur la COVID-19 par rapport aux vaccinations des enfants et adultes de routine et vaccination de rattrapage. Il est essentiel de renforcer la collecte de données pour mieux comprendre la couverture vaccinale chez différents groupes de migrants, âges et pays de la région MENA, en particulier pour les vaccins de rattrapage des immunisations de routine, et de développer des stratégies innovantes co-conçues pour répondre aux facteurs spécifiques d'adoption des vaccins identifies chez cette population.

**التغطية والاستفادة من التطعيم عند الأطفال والبالغين من المهاجرين واللاجئين في منطقة الشرق الأوسط وشمال أفريقيا: استعراض منهجي وتحليل تلوي**

أمنية بوعدي، فرح سيدات، حسن إدريس حسن محمد، إستيلا إفانجليدو، آنا ديل، آنا ريكينا مينديز، محمد خالص، سالي هارجريفز

**ملخص**

**الخلفية**

تضم منطقة الشرق الأوسط وشمال أفريقيا أكثر من 40 مليون مهاجر يعتبر المهاجرون واللاجئون فئة قد تكون غير محصنة بشكل كافٍ بسبب العوائق التي تواجههم في الحصول على الخدمات الصحية والتطعيمات في بلدان المنشأ أو العبور أو الوجهة. تهدف هذه الدراسة إلى تلخيص المعلومات المتعلقة بمستوى تقبل التطعيم وتغطية التطعيمات، والعوامل الاجتماعية والسلوكية المؤثرة والسياسات المرتبطة بالتطعيم للأطفال والبالغين المهاجرين في المنطقة.

**المنهجية**

قمنا بالبحث في ست قواعد بيانات، إلى جانب مواقع أخرى (مثل منظمة الصحة العالمية، منظمة الهجرة الدولية، وزارات الصحة) عن الدراسات المتعلقة بتغطية التطعيم، ومستوى قبول التطعيم، والدوافع الاجتماعية والسلوكية، والسياسات المتعلقة بتطعيم المهاجرين الأطفال والبالغين في منطقة الشرق الأوسط وشمال أفريقيا، بين عامي 2000 و27 أغسطس 2024 بجميع اللغات. تم تعريف المهاجرين كأفراد ينتقلون من مكان إقامتهم المعتاد، سواء داخل الحدود الدولية أو عبرها، بشكل مؤقت أو دائم. تم استبعاد الدراسات التي لا تحتوي على بيانات مفصلة للمهاجرين. كانت النتائج الرئيسية هي التغطية (نسبة الأفراد الذين تلقوا جرعة واحدة أو أكثر من أي لقاح) والقبول (نسبة الأفراد الذين قبلوا أي لقاح). تم جمع البيانات للأطفال (أقل من 18 عامًا) والبالغين (18 عامًا فأكثر) وتم تحليل البيانات بواسطة التحليل التلوي بنموذج التأثير العشوائي للنتائج المتجانسة، والنتائج التي لا يمكن إجراء التحليل التلوي عليها تم تلخيصها بشكل سردي. تم تلخيص عوامل تلقي التلقيح باستخدام نموذج الدوافع السلوكية والاجتماعية لمنظمة الصحة العالمية.

**النتائج**

شملت هذه الدراسة 55 ورقة علمية حول 1،906،975 مهاجرًا في 15 دولة (بما في ذلك اللاجئين في الشرق الأوسط). تم تقديم لقاح كورونا مجانًا للمهاجرين في جميع البلدان، بينما تم تقديم لقاحات الطفولة للمهاجرين في سبع دول. ومع ذلك، كانت هناك اختلافات في السياسات المتعلقة بالتطعيم للمراهقين والبالغين عبر الدول. كانت تغطية تطعيم الأطفال المهاجرين منخفضة، حيث تم تطعيم 36.0% فقط من 589 طفل مهاجر بشكل كامل حسب الجداول الوطنية (35.0%- 43.0%)، مما يشير إلى أن المهاجرين يواجهون مجموعة من العوائق التي تعيق إتمام التطعيم. بالنسبة للبالغين، وجدنا أن تغطية لقاح كورونا تراوحت بين 33.5% و84.8% للمهاجرين، و25.0% و59.0% للمواطنين. شملت دوافع عدم تلقي التطعيم لدى الأطفال محدودية توفر اللقاحات، وصعوبات مالية وإدارية، وقلة الوعي، وتخوفات الآباء. أما بالنسبة للبالغين، عدم تلقي لقاح كورونا كان سببه مخاوف متعلقة بأمان اللقاح وآثاره الجانبية وكذلك فقدان الثقة في النظام الصحي.

**الاستنتاجات**

يواجه المهاجرون عوامل خطر فريدة لعدم التحصين في منطقة الشرق الأوسط وشمال أفريقيا، وتعد مستويات التغطية منخفضة على الرغم من بعض الحقوق الممنوحة لهم فيما يتعلق بالاستفادة من خدمات التطعيم. البيانات المتوفرة حول التغطية والسياسات المتعلقة بالتطعيم لدى المهاجرين محدودة وصغيرة النطاق. هناك حاجة ماسة لتعزيز جمع البيانات لفهم التغطية بشكل أفضل عند مختلف الفئات العمرية والدول، وتطوير استراتيجيات مبتكرة لمعالجة العوامل الاجتماعية والسلوكية المؤثرة على تلقي التطعيم لدى هذه الفئة.
